# GNPS Untargeted GC‐MS Metabolomic Analysis of Essential Oils From *Duguetia lanceolata* and Evaluation of Antimicrobial Activity

**DOI:** 10.1002/cbdv.202503280

**Published:** 2026-02-25

**Authors:** Jackson Monteiro, Geovanna N. Antonelli, Erick P. Dantas, Simone S. Grecco, Marisi G. Soares, João Henrique G. Lago, Renata C. Pascon, Marcelo A. Vallim, Patricia Sartorelli

**Affiliations:** ^1^ Instituto De Ciências Ambientais, Químicas e Farmacêuticas Universidade Federal de São Paulo São Paulo Brazil; ^2^ Instituto Paulista de Resistência aos Antimicrobianos ‐ ARIES (CEPID FAPESP), Escola Paulista De Medicina – EPM UNIFESP São Paulo Brazil; ^3^ Instituto De Química Universidade Federal de Alfenas Alfenas Brazil; ^4^ Centro De Ciências Naturais e Humanas Universidade Federal do ABC Santo André Brazil

**Keywords:** antimicrobial activity, *Duguetia lanceolata*, essential oils, GNPS, untargeted GC‐MS metabolomic

## Abstract

In this study, the chemical composition of essential oils (EOs) extracted from leaves of *Duguetia lanceolata* collected in different seasons (autumn and winter) was analyzed using a GNPS untargeted GC‐MS metabolomic approach. EO from autumn leaves (EO‐A) contained 80 metabolites (93.8%), with limonene (28.5%), (+)‐β‐pinene (15.0%), cyclocolorenone (12.8%), 6,9‐guaiadiene (3.1%), and 4‐carvomenthenol (3.1%) as major constituents. EO from winter leaves (EO‐W) comprised 102 compounds (88.0%), dominated by β‐bisabolene (16.4%), β‐gurjunene (6.4%), β‐caryophyllene (4.7%), cyclocolorenone (3.9%), and dihydrocarveol (3.3%). The antimicrobial activity of both EOs was evaluated in vitro using the disk diffusion method against gram‐positive and gram‐negative bacteria, as well as selected yeast strains. MIC values ranged from 0.016 to 0.270 µg·mL^−^^1^, with EO‐A generally exhibiting lower MICs than EO‐W. EO‐A was active against *Acinetobacter baumannii*, *Candida krusei*, and *Candida parapsilosis*, whereas EO‐W showed no activity against these strains. Complementary in silico analyses supported these findings: PASS predictions suggested potential antibacterial and antifungal activity for the major constituents, and molecular docking indicated favorable binding of limonene and β‐bisabolene to DNA gyrase B (*A. baumannii*) and lanosterol 14α‐demethylase (CYP51, *C. albicans*), providing a mechanistic rationale for the observed antimicrobial effects. These results highlight the importance of seasonal collection in optimizing the bioactive potential of *D. lanceolata* leaves.

## Graphical abstract

Untargeted metabolomic analysis of the essential oil of *D. lanceolata* leaves collected in winter revealed that they are mostly composed of sesquiterpenes, while those collected in autumn showed a predominance of monoterpenes. Both essential oils displayed antimicrobial activity against bacterial and fungal species. All graphical elements were created using BioRender.com.

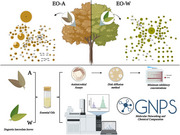

## 1 Introduction

Antimicrobial resistance (AMR) has become a growing problem worldwide, compromising the effectiveness of traditional antibiotics in the treatment of bacterial infections [1]. According to the World Health Organization (WHO), AMR is considered one of the biggest public health problems in the world. It is caused mainly by the disordered use of antimicrobial agents, leading to the death of millions of people annually and generating great economic losses for public organizations [2]. A group of bacteria that presents a huge potential for drug resistance mechanisms is the so‐called “ESKAPE,” comprising gram‐negative and gram‐positive bacteria that are the main cause of nosocomial infections. ESKAPE is the acronym of *Enterococcus faecium*, *Staphylococcus aureus*, *Klebsiella pneumoniae*, *Acinetobacter baumannii*, *Pseudomonas aeruginosa*, and *Enterobacter* species [3]. Although AMR refers especially to bacterial microorganisms, resistance also encompasses fungi and viruses, such as *Candida* species, HIV, and others [1].

Natural products are considered an important source of bioactive metabolites due to the huge chemodiversity and, consequently, range of biological activities. From the year 1981 to 2019, 162 drugs were approved to use as antibacterials, in which more than 50% originated from natural products or are semi‐synthetic derivative [4]. The presence of several compounds in the same extract confers a great advantage to natural products because they can act synergistically, being independently active through different mechanisms of action, increasing efficacy with minor possibilities to lead to antibacterial resistance [5]. In this context, essential oils (EOs), a complex natural extract from aromatic plants, have emerged as promising alternatives to available antimicrobial agents [6]. Essential oils demonstrate remarkable antimicrobial potential across a broad spectrum of pathogens. Beyond their intrinsic activity, their association with synthetic drugs represents a strategic approach to overcome a AMR, as EOs can enhance the bioactivity of conventional treatments [7, 8]. Among the commercially available species, the oils of lavender, thyme, peppermint, cajuput, tea tree, cinnamon, clove, eucalyptus, and sage are described with antimicrobial activity, including activity against multidrug‐resistant microorganisms and ESKAPE pathogens [3]. Furthermore, when discussing EOs with antimicrobial activity, the vast majority of studies focus on and are limited to the activities of well‐known aromatic species that are frequently used in daily life, in aromatherapy, and in the food and beverage industries, especially those cited previously [9]. Although many EOs have not yet been sufficiently explored, further studies are essential to understand their full potential in controlling infections in various contexts, such as clinical, food, and environmental. The antimicrobial activity of EOs occurs through different mechanisms, such as the destruction of bacterial cell membranes and their transport system, disruption of metabolic pathways, DNA synthesis inhibition, degradation of proteins, and the inhibition of protein synthesis [8]. A particularly relevant feature is their ability to inhibit the formation of biofilms, one of the main mechanisms of bacterial resistance. Bioactive compounds present in EOs, such as terpenes and phenolic compounds, show potential to combat resistance, interfering with both bacterial adhesion and proliferation within biofilms, making it difficult to develop persistent infections [10]. Considering their natural and multifaceted profile, EOs represent an innovative strategy in the fight against AMR, presenting themselves as a viable alternative for the development of new antimicrobial treatments. Therefore, the investigation of new species, both in chemical and pharmacological aspects, opens doors to the discovery of promising aromatic species that serve as sources of bioactive essential oils. In the context of natural products as a source of bioactive compounds, Brazil stands out. The country is considered the most biodiverse in the world, hosting over 20% of the species found globally, including more than 46 000 plant species [11, 12]. Biomes such as the Amazon, Atlantic Forest, and Cerrado are particularly notable. While the Amazon garners attention for its vast territorial size, the Atlantic Forest and Cerrado are recognized as biomes of high biodiversity, particularly endemic species at risk of loss, and are classified as biodiversity hotspot areas [13]. Thus, these regions urgently require actions aimed at their valorization and preservation. Found in subtropical America and widely distributed from the North to the South regions of Brazil, the genus *Duguetia* is considered the third largest genus of Annonaceae, after *Guateria* and *Annona*, and comprises approximately 100 species [14]. *D. lanceolata*, commonly known as “pindaíva” or “ateira‐da‐mata” [15], is native to subtropical America, and its occurrence has been reported in Ceará, Minas Gerais, Rio de Janeiro, São Paulo, Paraná, and Rio Grande do Sul States (Atlantic Forest and Cerrado biomes), although it is especially found in the Amazon biome [16]. In folk medicine, this plant has been used in the treatment of stomach, kidney, back pains [17], rheumatism, and as a sedative [18]. Previous studies reported the chemical composition of *D. lanceolata* essential oil from leaves, with a predominance of the sesquiterpenes *β*‐elemene, *β*‐selinene, caryophyllene oxide, and *β*‐eudesmol [19]. Also, the antinociceptive, anti‐inflammatory, and antimicrobial effects against *Staphylococcus aureus*, *Streptococcus pyogenes*, *Escherichia coli*, and *Candida albicans* of essential oils obtained from *D. lanceolata* barks were reported [20]. Furthermore, the antiaflatoxigenic and insecticidal properties of essential oil from leaves of *D. lanceolata*, mainly composed of *β*‐bisabolene and 2,4,5‐trimethoxystyrene, were evaluated, where the latter revealed a promising lethal effect against *Sitophilus zeamais* and *Zabrotes subfasciatus* [21]. Finally, as part of our continuous studies reporting the metabolic composition and antimicrobial activity evaluation of Brazilian plant essential oils, in this work, an untargeted metabolomic approach and the antimicrobial activity of essential oil, obtained from *D. lanceolata* during two different seasons in Brazil (autumn and winter), were exploited. To perform the untargeted metabolomic study of volatile metabolites, the data obtained from GC‐MS was analyzed using the unconventional molecular network approach, GNPS (Global Natural Products Social Molecular Networking), and, eventually, the NIST spectral database was used. Additionally, the in vitro antimicrobial activity of *D. lanceolata* essential oils against gram‐positive and gram‐negative bacteria and strains of some yeasts was evaluated, including *ESKAPE* and *Candida* microorganisms.

## 2 Results and Discussion

### 2.1 Untargeted Metabolomic—Metabolite Identification of Essential Oils

Leaves from *D. lanceolata* were collected during two different harvest seasons: autumn (EO‐A) and winter (EO‐W). After hydrodistillation, the obtained oils were analyzed by GC‐MS (Figure S1).

**FIGURE 1:**
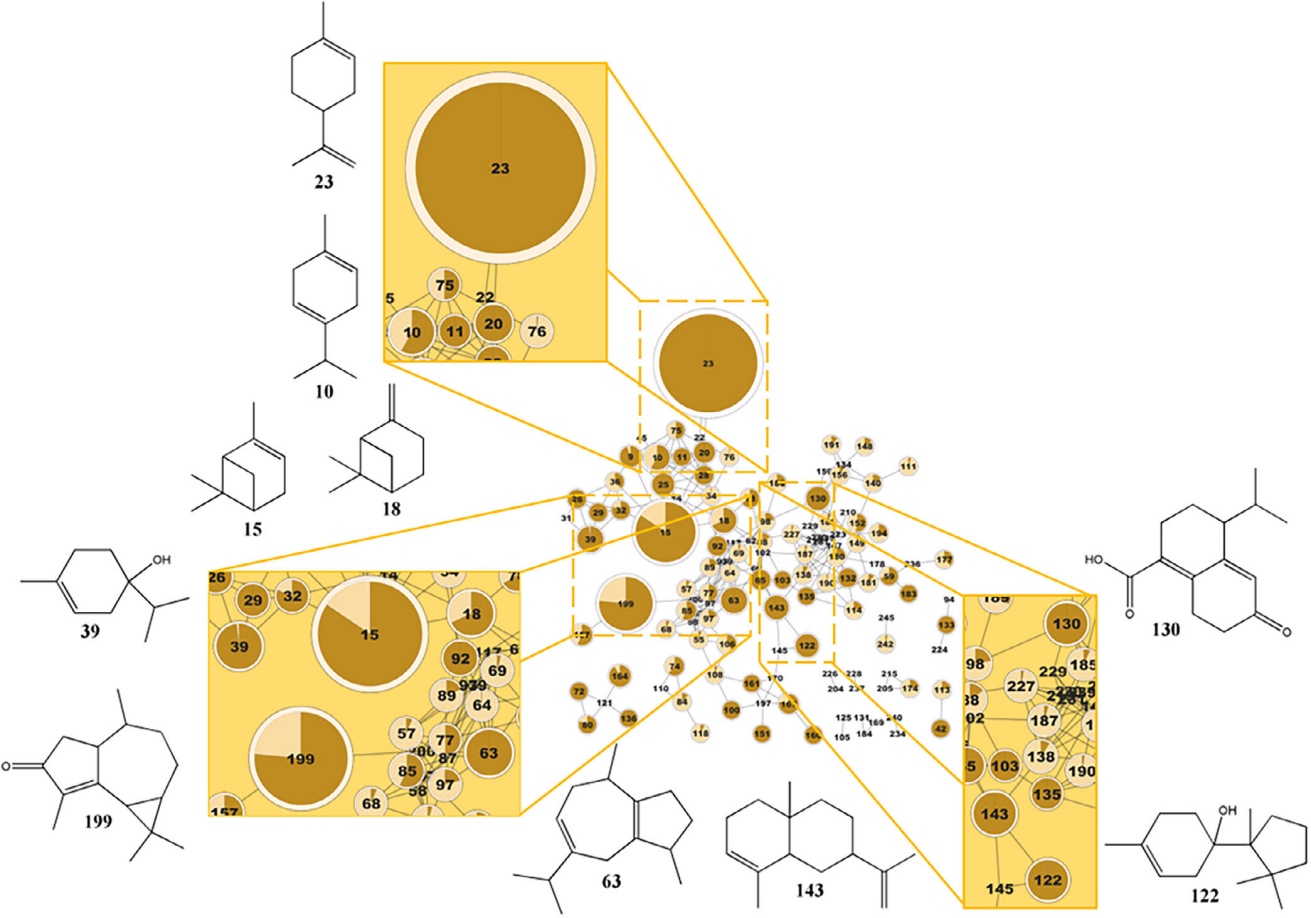
Molecular networking of GC‐MS data processed in GNPS, highlighting the monoterpene cluster identified in the EO‐A sample. Major annotated compounds include limonene (23), γ‐terpinene (10), α‐pinene (15), β‐pinene (18), 4‐carvomenthenol (39), 6,9‐guaiadiene (63), cuprenen‐1‐ol <4‐> (122), khusinol (130), α‐selinene (143), and cyclocolorenone (199).

**FIGURE 2:**
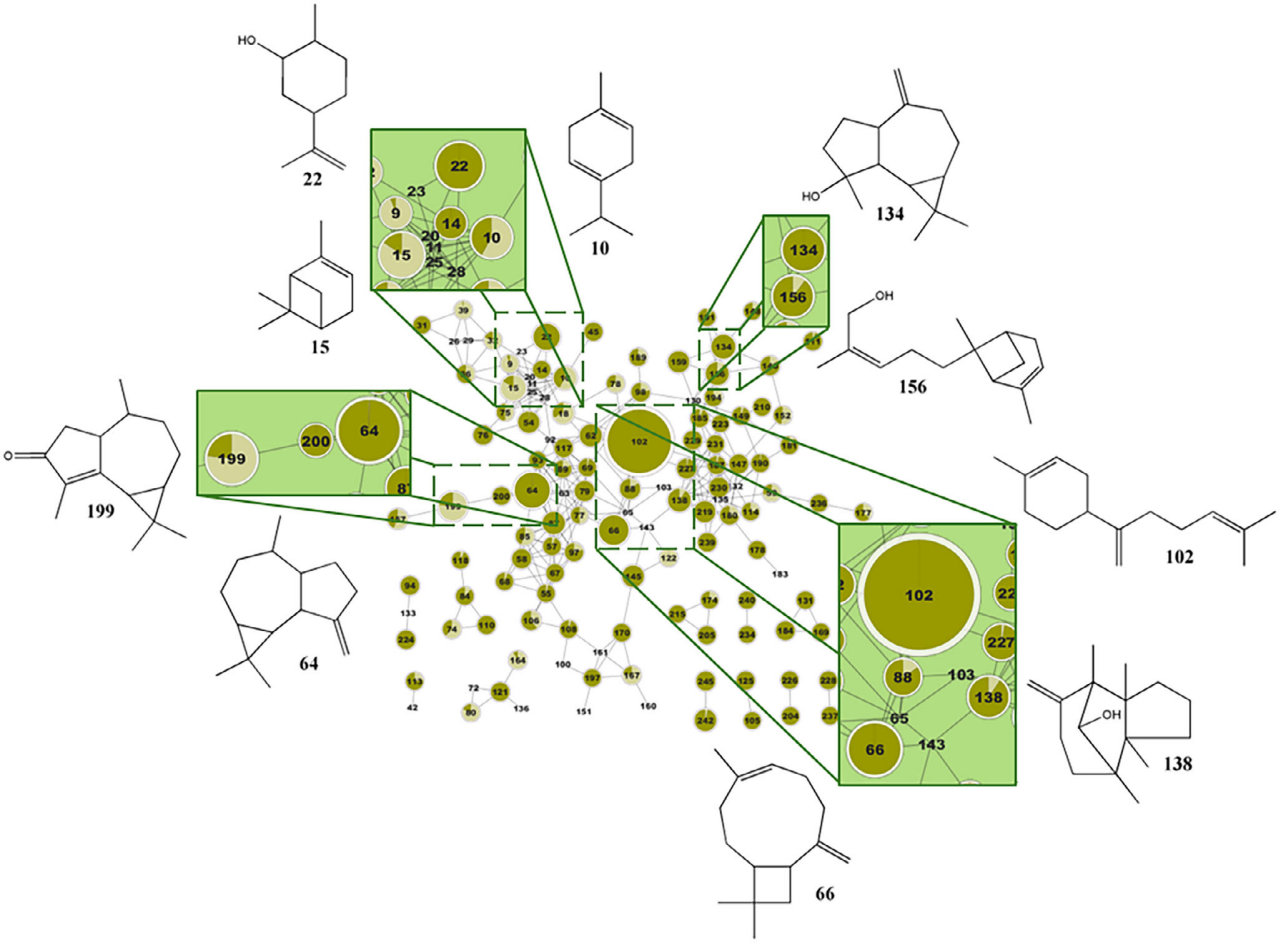
Molecular networking of GC‐MS data processed in GNPS, highlighting the sesquiterpene cluster identified in the EO‐W sample. Major annotated compounds include γ‐terpinene (10), α‐pinene (15), dihydrocarveol (22), β‐gurjunene (64), β‐caryophyllene (66), β‐bisabolene (102), spathulenol (134), gymnomitrol (138), (Z)‐bergamotol (156), and cyclocolorenone (199).

The GNPS platform, together with other databases such as NIST, MassBank, and ReSpect, among others, contains over 1 164 920 MS/MS spectra and at least 18,163 substances, facilitating, in part, the annotation of metabolites [22, 23]. Furthermore, it is still required to manually analyze some mass spectra of interest when consulting databases such as SciFinder and the Dictionary of Natural Products. The molecular networking was built using the GNPS platform to explore the differences in volatile constituents between the two oils (Figure 2). By comparing the metabolic profiles of each essential oil, the network generated 126 nodes (Figures 1 and 2). Node annotation was performed using the GNPS library in conjunction with the NIST 08 database and fragmentation data acquired via GC‐MS. It is also worth mentioning that few articles are available in the literature using GNPS to perform annotation of plant essential oil metabolites and the construction of molecular networks.

**FIGURE 3:**
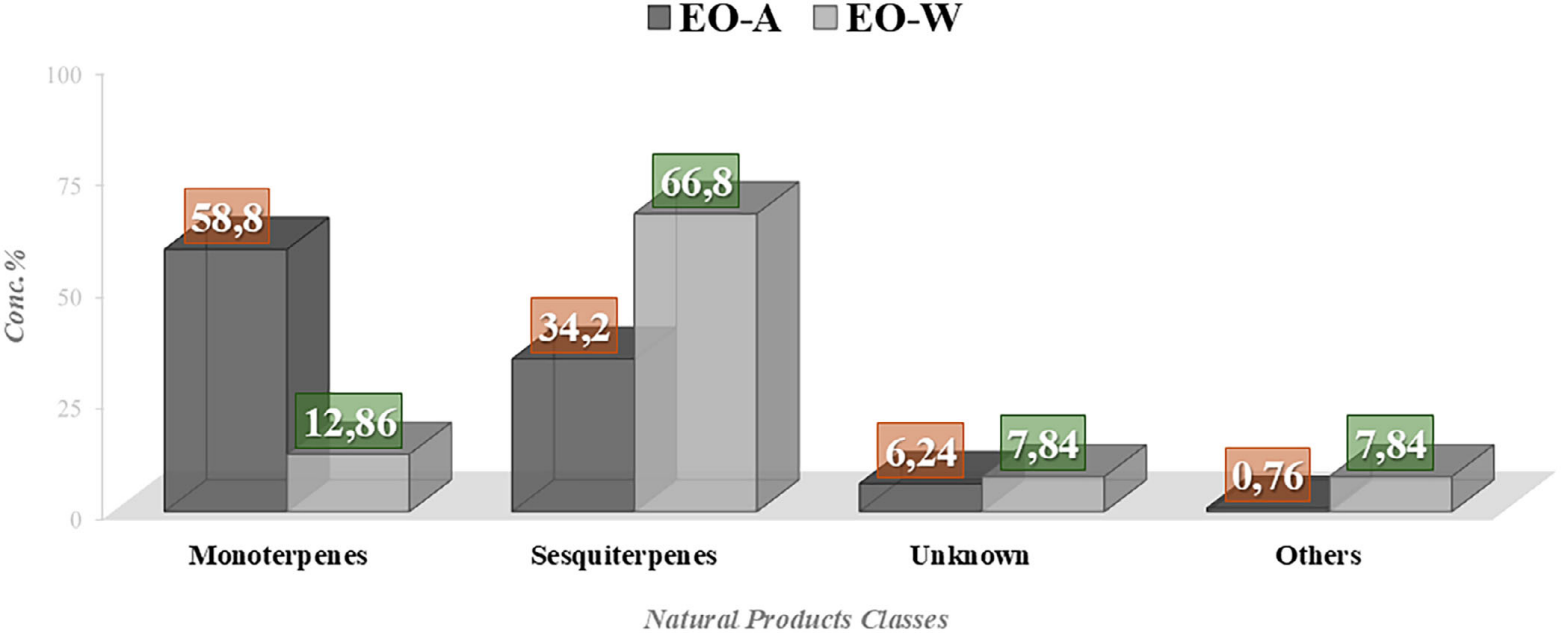
Classes of major compounds identified in the chemical composition of *D. lanceolata* essential oil collected in winter (EO‐W) and autumn (EO‐A).

According to the GC‐MS and GNPS analysis results (Table S1), 78 metabolites were annotated in EO‐A, accounting for 93.8% of its total composition. The untargeted metabolomic analysis revealed the presence of limonene (28.5%), α‐pinene (15.1%), cyclocolorenone (12.8%), 6,9‐guaiadiene (3.1%), 4‐carvomenthenol (3.1%), γ‐terpinene (2.8%), β‐pinene (2.8%), α‐selinene (2.4%), khusinol (2.2%), and cuprenen‐1‐ol (2.1%). In EO‐W, 99 metabolites were annotated, representing 87.5% of its composition. The main compounds identified were β‐bisabolene (16.4%), β‐gurjunene (6.39%), β‐caryophyllene (4.7%), cyclocolorenone (3.9%), dihydrocarveol (3.3%), α‐pinene (2.8%), spathulenol (2.4%), (Z)‐bergamotol (2.1%), gymnomitrol (2.0%), and γ‐terpinene (2.0%). In EO‐A, monoterpenes (58.8%) were the predominant class, followed by sesquiterpenes (34.2%). Conversely, EO‐W was mainly composed of sesquiterpenes (66.8%) and monoterpenes (12.9%) (Figure 3). This seasonal variation reflects the distinct chemical profiles of the oils: many of the major compounds found in EO‐A were absent in EO‐W, and vice versa.

According to the literature, essential oils from different genera of the Annonaceae family are primarily composed of mono‐ and sesquiterpenes, especially those with menthane (limonene and *p*‐cymene), pinane (*α*‐ and *β*‐pinene), and caryophyllane (*β*‐caryophyllene and caryophyllene oxide) skeletons [24]. Across several Annonaceae genera, including *Annona*, *Anaxagorea*, *Fusaea*, *Xylopia*, *Guatteria*, *Hexalobus*, and *Pachypodanthium*, compounds such as bicyclogermacrene, spathulenol, germacrene D, caryophyllene oxide, α‐pinene, and β‐caryophyllene are considered chemophenetic markers [25, 26, 27, 28, 29, 30]. Between 2011 and 2021, 49 studies reported the identification of nearly 100 chemical compounds from essential oils of Brazilian Annonaceae species, with *Annona* and *Xylopia* (11 studies each), *Guatteria* (9), and *Duguetia* (7) being the most studied genera. The most frequently reported compounds include α‐pinene, β‐pinene, limonene, (E)‐caryophyllene, bicyclogermacrene, caryophyllene oxide, germacrene D, spathulenol, and *β*‐elemene [24]. EOs from various parts of *D. furfuracea*, *D. lanceolata*, *D. quitarensis*, and *D. gardneriana* revealed sesquiterpenes as the predominant class. Studies on the essential oil from the bark of *D. lanceolata* showed that, within the first 2 h of extraction, β‐elemene, caryophyllene oxide, and β‐selinene were the major constituents [20]. Similarly, EO extracted from branches/twigs contained the same major sesquiterpenes [31]. Ribeiro et al. identified β‐bisabolene and trimethoxystyrenes in leaf EO from a *D. lanceolata* species collected in São Paulo, while β‐selinene, aristolochene, (*E*)‐caryophyllene, and (*E*)‐calamenene were identified in specimens from Minas Gerais [21]. Likewise, β‐selinene, aristolochene, (*E*)‐caryophyllene and (*E*)‐calamenene were identified in leaves of specimens collected in Minas Gerais [32]. Recent studies have enabled the identification of a higher number of oil constituents and allowed evaluation of seasonal variation in chemical composition. Abiotic factors such as season, location, climate, soil type, water stress, and isolation methods can significantly affect the chemical makeup and biological activity of essential oils [33, 34, 35].

### 2.2 Antimicrobial Activity

#### 2.2.1 Disk Diffusion Method

**FIGURE 4:**
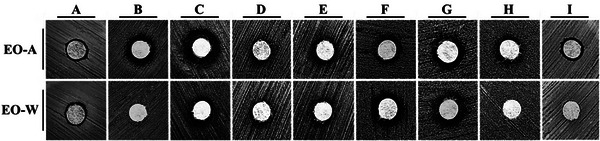
Inhibition halos demonstrating the antimicrobial activity of *D. lanceolata* essential oils via the disk diffusion method. Rows correspond to seasonal samples: **EO‐A (autumn)** and **EO‐W (winter)**. Columns indicate the tested strains: (A) *Staphylococcus aureus* (ATCC 25923); (B) *Acinetobacter baumannii* (ATCC 19606); (C) *Saccharomyces cerevisiae* (BY4647); (D) *Cryptococcus neoformans* (KN99α, serotype A); (E) *C. neoformans* (JEC21, serotype D); (F) *Cryptococcus gattii* (R265, serotype B); (G) *C. gattii* (NIH312, serotype C); (H) *Candida krusei* (clinical isolate 9602); and (I) *Candida parapsilosis* (clinical isolate 68).

**FIGURE 5:**
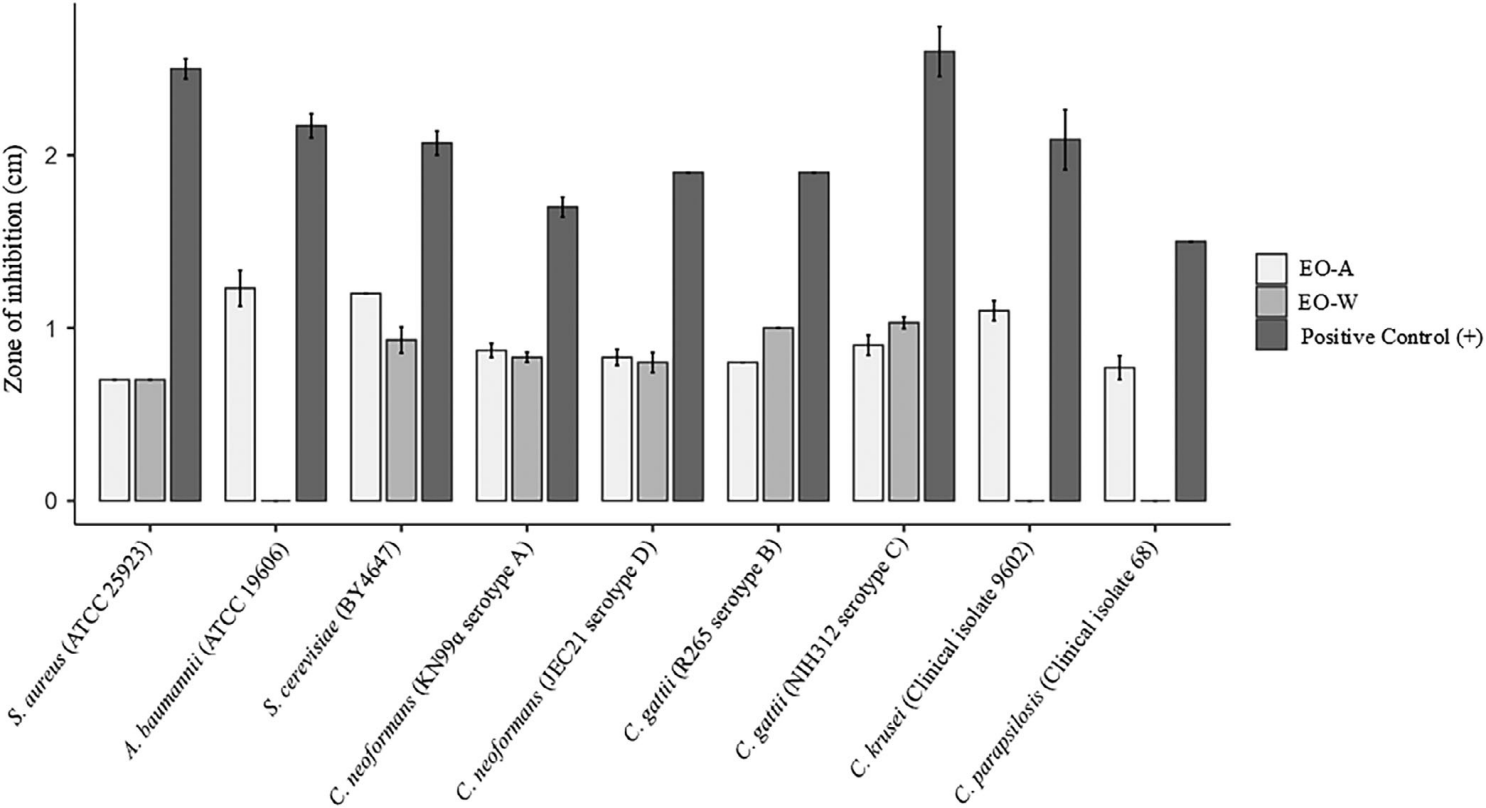
Disk diffusion method. **(+)**: Positive control consisting of **kanamycin**—*S. aureus* (ATCC25923) and *A. baumanii* (ATCC 19606); **eugenol**—*S. cerevisiae* (BY4647), *C. neoformans* (KN99α—sorotipe A and JEC21—sorotipe D), *C. gatti* (R265—sorotipe B and NIH312—sorotipe C) and *C. krusei* (isolado clinico 9602); **amphotericin B**—*C. parapsilosis* (isolado clinico 68). Statistical graphics were produced using jamovi software.

The antimicrobial activity of the samples was initially evaluated using the disk diffusion assay, which was applied as a qualitative screening method to compare relative microbial susceptibility. Because this technique relies on compound diffusion through solid media, inhibition zones were interpreted comparatively rather than as quantitative indicators of potency (Figures 4, 5 and Tables S2–S5).

Against *Staphylococcus aureus* (ATCC 25923), both samples produced small and identical inhibition zones (0.70 cm), which were significantly smaller than the positive control (2.50 ± 0.10 cm; *p* < 0.001), indicating limited antibacterial activity under agar diffusion conditions. For *Acinetobacter baumannii* (ATCC 19606), EO‐A showed a measurable inhibition zone (1.23 ± 0.18 cm), whereas EO‐W showed no detectable activity (0.00 cm); both treatments differed significantly from the positive control (2.17 ± 0.12 cm; *p* < 0.01), suggesting selective activity associated with EO‐A.

In *Saccharomyces cerevisiae* (BY4647), inhibition zones of 1.20 cm (EO‐A) and 0.93 ± 0.13 cm (EO‐W) were observed, both significantly smaller than the control (2.07 ± 0.12 cm; *p* < 0.01), indicating moderate antifungal effects. For *Cryptococcus neoformans* (serotypes A and D), both treatments produced small but consistent inhibition zones ranging from 0.80 to 0.87 cm, significantly lower than those of the positive controls (1.70–1.97 cm; *p* < 0.01). Similar trends were observed for *Cryptococcus gattii* (serotypes B and C), with inhibition zones between 0.80 and 1.03 cm, whereas positive controls reached up to 2.60 cm (*p* < 0.05).

For *Candida krusei* and *Candida parapsilosis*, EO‐A exhibited inhibition zones of 1.10 ± 0.10 cm and 0.77 ± 0.12 cm, respectively, while EO‐W showed no detectable activity. In both cases, the inhibition zones were significantly smaller than those of the positive controls (1.50–2.09 cm; *p* < 0.05).

According to Sharma and collaborators, terpenoids, especially mono‐ and sesquiterpenes, act as natural antimicrobial and antifungal compounds protecting the plant itself. The author also described the mechanisms of action of terpenes as antimicrobial agents, such as cell membrane disruption, anti‐quorum sensing action, and nucleic acid and protein inhibition mechanisms, highlighting the hydrophobicity of terpenoids and their potential to diffuse through the lipid bilayer of the bacterial cell membrane and lower their ability to disrupt the lipopolysaccharide outer membrane of the GN bacteria [36]. Recent studies have highlighted the potential of essential oils to combat *A. baumannii*, a multi‐resistant pathogen responsible for hospital infections and capable of forming biofilms, which makes treatment with conventional antimicrobials difficult. Essential oils such as *Eucalyptus camaldulensis, Myrtus communis, Cinnamomum verum* (cinnamon), and *Litsea cubeba* showed strong antimicrobial activity, all of which have monoterpenes in their composition, showing efficacy against both planktonic cells and biofilms of *A. baumannii* [37]. *Litsea cubeba* oil (LCEO), in turn, showed strong antibacterial action, affecting cell membrane permeability, causing damage to bacterial morphology, and interfering with protein synthesis [38]. In addition, the research also explored the interaction of these essential oils with conventional antibiotics, resulting in synergistic effects, which could enhance the fight against resistant strains. These results indicate that essential oils, which mostly contain monoterpenes, may be promising in combination with traditional therapies, offering new approaches to controlling hospital infections caused by *A. baumannii* [39].

Overall, the disk diffusion results indicate discrete but reproducible antimicrobial effects, with clear differences among microorganisms and between samples. However, the generally small inhibition zones and the absence of activity in some cases highlight the limitations of agar diffusion for complex matrices, reinforcing that this assay served primarily as a qualitative screening step. These findings justified subsequent evaluation using the MIC assay, which provided a quantitative and more reliable assessment of antimicrobial potency.

#### 2.2.2 Minimum Inhibitory Concentrations (MIC)

**FIGURE 6:**
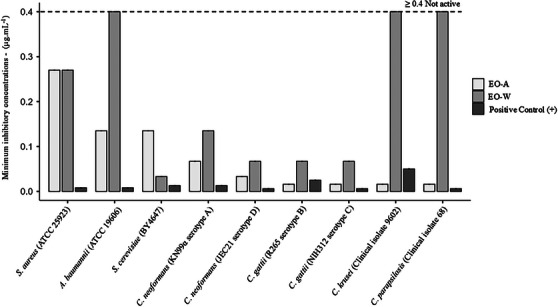
Minimum inhibitory concentrations obtained by broth microdilution sensitivity assay. Statistical graphics were produced using jamovi software.

The antimicrobial potential of the samples was first evaluated using the disk diffusion assay as a qualitative screening method, followed by determination of the minimum inhibitory concentration (MIC_90_) to provide a quantitative assessment of activity (Figure 6 and Tables S6–S8).

Following the qualitative screening by disk diffusion, the antimicrobial activity of the samples was quantitatively evaluated by determination of the MIC_90_. This assay provides a diffusion‐independent measurement of antimicrobial potency and is therefore more appropriate for comparing complex natural matrices.

Both samples exhibited measurable antibacterial activity against *S. aureus* (ATCC 25923), with identical MIC_90_ values of 0.270 µg mL^−^^1^. Statistical analysis confirmed significant differences between the samples and the positive control (*p* < 0.001), while no significant difference was observed between EO‐A and EO‐W, consistent with their comparable qualitative profiles.

In contrast, a marked difference was observed for *A. baumannii* (ATCC 19606). EO‐A showed a MIC_90_ of 0.135 µg mL^−^^1^, whereas EO‐W exhibited substantially reduced activity (MIC_90_ ≥ 0.400 µg mL^−^^1^). One‐way ANOVA followed by Tukey's post hoc test revealed highly significant differences among treatments (*p* < 0.001), indicating selective antibacterial activity associated with EO‐A against this gram‐negative pathogen.

The antifungal activity was more pronounced across all tested species. For *S. cerevisiae* (BY4647), MIC_90_ values of 0.135 µg mL^−^^1^ (EO‐A) and 0.033 µg mL^−^^1^ (EO‐W) were obtained, with statistically significant differences relative to the positive control (*p* < 0.001). Within the *Cryptococcus* complex, consistently low MIC_90_ values were observed, particularly for *C. gattii* serotypes B and C, for which EO‐A exhibited MIC_90_ values of 0.016 µg mL^−^^1^. Similar trends were detected for *C. neoformans* serotypes A and D, confirming robust antifungal activity.

For *Candida* species, EO‐A again demonstrated superior activity, with MIC_90_ values of 0.016 µg mL^−^^1^ against both *C. krusei* and *C. parapsilosis*. In contrast, EO‐W showed substantially weaker or no detectable activity (MIC_90_ ≥ 0.400 µg mL^−^^1^). All comparisons among treatments were statistically significant according to ANOVA and Tukey's post hoc test (*p* < 0.001).

The varying MICs observed for each of the oils may be attributed to the composition and concentration of the compounds present in them. In EO‐W, the following classes of constituents were identified: sesquiterpenes (66.8%), monoterpenes (12.9%), and other compounds (10.1%), with sesquiterpenes being the dominant class (Figure 4). On the other hand, EO‐A contained the following compound classes: monoterpenes (58.8%), sesquiterpenes (34.2%), and other compounds (0.7%). While the oil obtained in autumn showed a higher concentration of monoterpenes, the oil obtained in winter demonstrated a higher concentration of sesquiterpenes.

Although sesquiterpenes predominate in EO‐W and monoterpenes in EO‐A, the specific metabolites responsible for the antibacterial activity in either essential oil remain unclear. Wiles, Pearson, and Beddoe highlight the antibacterial and antibiofilm properties of terpenes against gram‐positive strains, including limonene, *β*‐caryophyllene, and α‐pinene as promising candidates. The authors also emphasize the ability of this class of compounds to combat antibiotic resistance, as they can antagonize hydrolase enzymes in gram‐positive strains and disrupt the lipopolysaccharide layer and cytoplasmic membrane of gram‐negative bacteria, both of which are involved in bacterial resistance [40, 41].

Nevertheless, it is likely that the actions of these components are also influenced by synergistic effects. Therefore, further investigation of essential oils as supplementary antibacterial agents is warranted. The more promising antimicrobial activity of EO‐A, compared to EO‐W, can be explained by the lipophilic nature of the monoterpenes predominantly found in EO‐A. Sikkema et al. observed that the effect of cyclic hydrocarbons on the structural and functional properties of biological membranes was directly related to the accumulation of these compounds in the membranes, which led to a loss of membrane integrity [42]. Cristani et al. proposed that the antibacterial activity of the monoterpenes investigated (thymol, carvacrol, *p*‐cymene, and γ‐terpinene) was due, at least in part, to their ability to penetrate and disrupt the lipid fraction of plasma membranes, thereby compromising their permeability. These compounds were also shown to interact with intracellular components essential for antibacterial action [43].

The most common terpene compounds found in essential oils are monoterpenes, which account for around 90% of volatile oils, followed by sesquiterpenes [44]. However, despite the numerous studies on sesquiterpenes, especially oxygenated sesquiterpenes, no research has identified antimicrobial activity in essential oils, whose majority compounds were similar to those in this study [45, 46].

#### 2.2.3 In Silico Prediction of Biological Activity (PASS‐Prediction of Activity Spectra)

To investigate the molecular basis underlying the differential antimicrobial activity observed between the essential oils, the biological activity spectra of their major constituents were predicted using the PASS Online platform [47]. The theoretical analysis (Table 1) revealed a moderate probability of biological action for most compounds, with Pa (probability to be active) values predominantly ranging from 0.3 to 0.6 for both antibacterial and antifungal activities. These values are consistent with the structural simplicity and high hydrophobicity of monoterpenes and sesquiterpenes, which typically exhibit broad but moderate bioactivity profiles in in silico screenings [48, 49].

**TABLE 1:** Chemical composition and predicted biological activity spectra (PASS) of the major compounds identified in autumn (EO‐A) and winter (EO‐W) essential oils.

Class/compound	EO‐A (%)	EO‐W (%)	Pa antibacterial	Pa antifungal
*Monoterpenes*				
Limonene	28.53	—	0.405	0.582
α‐Pinene	15.06	2.78	0.326	0.439
4‐Carvomenthenol	03.09	0.06	0.328	0.466
γ‐Terpinene	2.83	02.04	0.325	0.443
β‐Pinene	2.82	1.33	0.233	0.225
Dihydrocarveol	—	3.26	0.507	0.664

*Sesquiterpenes*				
β‐Bisabolene	—	16.45	0.413	0.585
Cyclocolorenone	12.78	3.92	0.260	0.364
β‐Gurjunene	0.01	6.39	0.411	0.482
β‐Caryophyllene	—	4.72	0.437	0.582
6,9‐Guaiadiene	3.15	—	0.260	0.470
α‐Selinene	2.44	—	0.355	0.548
Spathulenol	—	2.43	0.408	0.519
Khusinol	2.23	—	0.401	0.388
Gymnomitrol	0.21	2.05	0.404	0.371
(z)‐Bergamotol	0.24	2.12	0.430	0.454
Cuprenen‐1‐ol	2.06	0.01	0	265

*Note*: Compounds are grouped by chemical class and listed in descending order of concentration. Values represent the relative percentage area. Pa > 0.7 indicates high probability; 0.5 < Pa < 0.7 indicates moderate probability.

Abbreviation: Pa = probability to be active.

Remarkably, limonene, the major constituent of the biologically active EO‐A, and β‐bisabolene, the predominant compound in the less active EO‐W, were assigned nearly identical Pa values. For antibacterial activity, both compounds exhibited Pa ≈ 0.41, while for antifungal activity, similarly close values were obtained (Pa ≈ 0.58). However, these theoretical predictions contrast sharply with the experimental MIC data, in which EO‐A demonstrated markedly superior efficacy, not only against *Acinetobacter baumannii* but also against yeasts of the *Cryptococcus* and *Candida* genera.

This divergence between in silico predictions based on 2D molecular descriptors and the experimentally observed biological performance highlights an important limitation of global activity predictors such as PASS. While effective for identifying broad pharmacological tendencies, these models do not capture critical determinants of antimicrobial potency, including stereochemical complementarity, three‐dimensional (3D) recognition at specific molecular targets, membrane‐partitioning behavior, and differential bioavailability within microbial cells [50].

Therefore, the comparable Pa values assigned to limonene and β‐bisabolene suggest that the pronounced superiority of EO‐A cannot be attributed solely to the intrinsic activity of its major compound as inferred from structural class. Instead, the results support the hypothesis that antimicrobial efficacy in these essential oils arises from more specific molecular interactions and pharmacokinetic factors, potentially involving selective binding to microbial targets, differences in membrane permeability, and synergistic effects among minor constituents. This interpretation provides a strong rationale for the subsequent application of molecular docking and ADME/T analyses, aimed at elucidating the mechanisms of selectivity, target engagement, and bioavailability that underlie the experimentally observed antimicrobial profiles [51].

#### 2.2.4 Molecular Docking

Molecular docking simulations were performed against *A. baumannii* DNA gyrase (PDB: 7PQL) and *C. albicans* CYP51 (PDB: 5FSA) to provide a mechanistic interpretation for the distinct antimicrobial profiles observed for EO‐A and EO‐W [52, 53, 54].

For the antifungal evaluation, the *C. albicans* CYP51 structure was selected as a surrogate model to rationalize the activity observed against *C. krusei* and *C. parapsilosis*. Although *C. albicans* was not explicitly tested in vitro, this approach is scientifically grounded on the high structural orthology of the CYP51 active site across the *Candida* genus, the robustness of the available crystallographic data, and the validated mechanism of the positive control (fluconazole), allowing for a reliable extrapolation of the binding modes [55].

**FIGURE 7:**
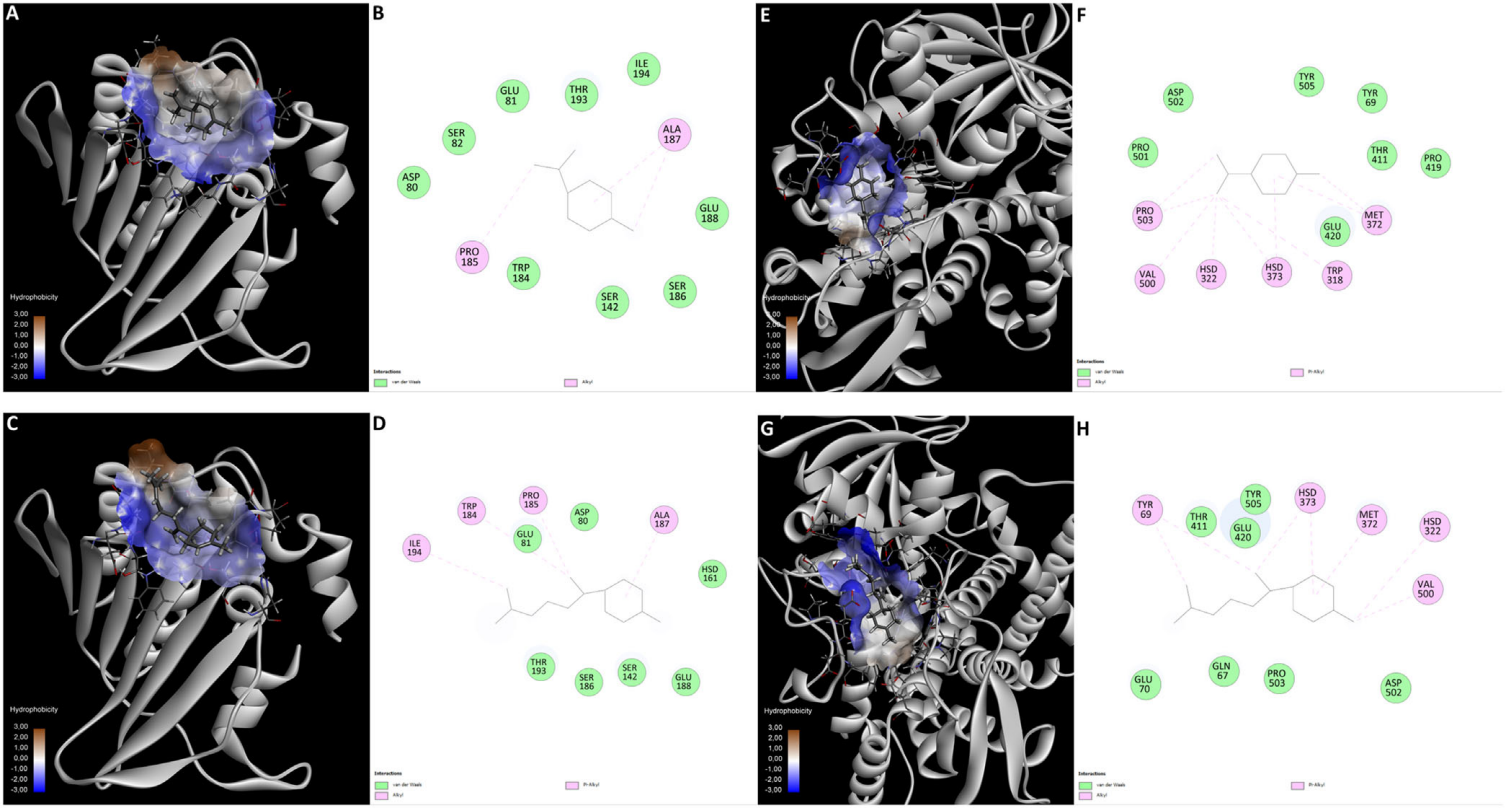
Molecular docking analysis of the major terpenes from *D. lanceolata* essential oils against bacterial and fungal targets. (A–B) Limonene (major compound of the active EO‐A) docked into the ATPase domain of *A. baumannii* DNA Gyrase B (PDB: 7PQL). (A) 3D binding mode showing the accommodation of limonene within a hydrophobic cleft of the enzyme. (B) 2D interaction diagram highlighting predominant alkyl and π–alkyl contacts with PRO185 and ALA187, surrounded by a van der Waals cage formed by ASP80, GLU81, SER82, and TRP184. (C–D) β‐bisabolene (the major compound of the inactive EO‐W) docked into the same DNA gyrase B site. (C) 3D binding mode illustrating the extension of the aliphatic chain along a hydrophobic groove. (D) 2D interaction diagram showing Alkyl contacts with PRO185, ALA187, TRP184, and ILE194, but with reduced steric complementarity and overall weaker stabilization. (E–F) Limonene docked into lanosterol 14α‐demethylase (CYP51) from *Candida albicans* (PDB: 5FSA). (E) 3D binding mode depicting insertion into the deep hydrophobic channel leading to the heme‐containing active site. (F) 2D interaction diagram evidencing a dense network of alkyl and π–alkyl interactions with VAL500, PRO503, MET372, and HSD322, supported by the aromatic environment formed by TYR69 and TYR505. (G–H) β‐Bisabolene docked into CYP51. (G) 3D binding mode showing partial accommodation within the catalytic pocket with an extended conformation. (H) 2D interaction diagram revealing predominance of weaker van der Waals contacts (e.g., GLU70, GLN67, PRO503) and fewer strong hydrophobic anchors, consistent with a looser fit and reduced binding efficiency.

Ideally, molecular docking scores are expected to correlate with biological activity; however, such correlations are not mandatory in complex biological systems, particularly when pharmacokinetic and physicochemical factors dominate and are not captured by thermodynamic docking models. In the present study, docking analyses revealed comparable interaction energies for limonene and β‐bisabolene toward both evaluated targets. Against the ATPase domain of *A. baumannii* DNA gyrase B, limonene exhibited a binding energy of Δ*G* = −5.72 kcal mol^−^^1^, while β‐bisabolene showed a slightly more favorable value (Δ*G* = −5.96 kcal mol^−^^1^) (Figure 7A,B). Structurally, limonene was accommodated within a compact hydrophobic cleft, stabilized mainly by alkyl and π–alkyl interactions with PRO185 and ALA187, and supported by a surrounding van der Waals environment formed by ASP80, GLU81, SER82, and TRP184. In contrast, β‐bisabolene extended along a hydrophobic groove, engaging additional alkyl contacts with TRP184 and ILE194, although with reduced steric complementarity (Figure 7C,D).

Similarly, docking to lanosterol 14α‐demethylase (CYP51) from *Candida albicans* resulted in Δ*G* values of −5.99 kcal mol^−^^1^ for limonene and −6.48 kcal mol^−^^1^ for β‐bisabolene. Limonene occupied the deep hydrophobic channel leading to the heme‐containing active site, forming a dense network of alkyl and π–alkyl interactions with VAL500, PRO503, MET372, and HSD322, further stabilized by the aromatic environment provided by TYR69 and TYR505 (Figure 7E,F). Conversely, β‐bisabolene adopted a more extended conformation within the catalytic pocket, relying predominantly on weaker van der Waals interactions with residues such as GLU70, GLN67, and PRO503, consistent with a less compact fit (Figure 7G,H).

Despite these modest energetic differences favoring β‐bisabolene in silico, experimental MIC assays consistently demonstrated superior antimicrobial activity for the limonene‐rich oil (EO‐A). This apparent discrepancy suggests that the enhanced activity of EO‐A is more plausibly attributed to whole‐cell effects rather than stronger target‐specific binding affinity alone. As a small and highly lipophilic monoterpene (C_10_), limonene is expected to permeate fungal cell walls and bacterial membranes more efficiently than the bulkier sesquiterpene β‐bisabolene (C_15_), achieving higher effective intracellular concentrations. Furthermore, monoterpenes are widely reported to disrupt membrane integrity through non‐specific interactions with lipid bilayers, promoting permeability changes and cellular stress that may act synergistically with enzymatic inhibition but are not captured by receptor‐centered docking simulations [56, 57]. In contrast, although β‐bisabolene is capable of interacting with enzymatic targets, its larger and more flexible scaffold may limit intracellular accessibility, ultimately resulting in reduced antimicrobial efficacy in MIC‐based whole‐cell assays.

Although PASS predictions initially assigned similar antimicrobial probabilities to both terpenes, structural docking analysis revealed relevant differences in target engagement at the binding‐site level. Limonene exhibited a more compact and sterically efficient accommodation within hydrophobic cavities of DNA gyrase and formed denser hydrophobic interaction networks within the CYP51 catalytic channel, whereas β‐bisabolene adopted more extended conformations and relied predominantly on weaker van der Waals contacts [56]. These qualitative differences in binding organization, combined with the superior cellular accessibility of limonene, provide a coherent molecular rationale for the enhanced antimicrobial efficacy observed for the limonene‐rich oil (EO‐A) in whole‐cell assays [52, 58].

#### 2.2.5 ADME/T (Pharmacokinetics)

To assess the translational potential of the bioactive terpenes, physicochemical and pharmacokinetic properties were predicted using the SwissADME platform (Figures S2–S3) [59]. The analysis of drug‐likeness (Lipinski's Rule of Five) revealed that limonene satisfies all criteria (0 violations), while β‐bisabolene presents only one violation related to lipophilicity (MLOGP > 4.15).

Both terpenes exhibited limited aqueous solubility (ESOL and Ali classes ranging from “moderately soluble” to “poorly soluble”), which is typical for hydrophobic essential oil constituents and suggests that formulation strategies (e.g., nanoemulsions) would be required for pharmaceutical development. Nevertheless, both compounds achieved a bioavailability score of 0.55, suggesting adequate structural features for potential therapeutic candidates [60].

The “Boiled‐Egg” permeation model (Figure S4) highlighted distinct distribution profiles between the marker compounds [61]. Limonene is located within the yolk (yellow region), indicating a high probability of blood–brain barrier (BBB) permeation. This characteristic is consistent with its lower molecular weight (136.23 g mol^−1^) and lipophilic nature, suggesting potential utility in treating systemic or central nervous system fungal infections. In contrast, β‐bisabolene falls outside the absorption ellipse, indicating restricted central access and a distribution likely confined to peripheral tissues.

Regarding metabolic safety, the inhibitory potential against five major human cytochrome P450 isoforms (CYP1A2, CYP2C9, CYP2C19, CYP2D6, and CYP3A4) was evaluated to predict the risk of drug‐drug interactions. The probabilistic analysis (Figure S5) suggests a generally favorable safety profile. For CYP3A4, the enzyme responsible for metabolizing the majority of clinical drugs, limonene and β‐bisabolene displayed inhibition probabilities of 0.49 and 0.42, respectively, remaining below the threshold of high risk [59, 62].

Although the qualitative classification flagged a potential interaction with CYP2C9 for both compounds, the quantitative probability scores for this isoform were relatively moderate (0.26 for limonene and 0.34 for β‐bisabolene). Collectively, these findings highlight a selectivity window: while the molecular docking results demonstrated strong binding affinity toward the fungal CYP51 (essential for pathogen survival), the ADMET predictions suggest a lower propensity to inhibit the human hepatic cytochrome system, minimizing the risk of host toxicity [62].

### 2.3 Biological Relevance and Therapeutic Perspectives

Regarding the activity of the essential oils against yeast microorganisms, particular attention should be given to the inhibitory effects of *D. lanceolata* oils on *Candida* spp., as fungi from this genus are clinically relevant opportunistic pathogens and frequently exhibit intrinsic or acquired resistance to antifungal agents. Notably, EO‐A demonstrated activity against a clinical isolate of *C. krusei*, a species recognized for its intrinsic resistance to several azole antifungals [63]. This finding is especially relevant and may be directly associated with the presence of limonene in EO‐A. Limonene has been reported as a promising antifungal agent capable of attenuating fungal virulence by inhibiting growth, adhesion, and key morphological transitions, including hyphal and filament formation in *Candida* spp. [64].

Overall, the MIC_90_ data indicate that the essential oil samples exhibit moderate antibacterial activity but pronounced antifungal potential, with EO‐A consistently outperforming EO‐W. The statistically significant differences observed among the tested microorganisms confirm the robustness of the assay and validate MIC determination as a reliable quantitative complement to the qualitative disk diffusion screening. These results reinforce the notion that seasonal variation in essential oil composition translates directly into biologically meaningful differences in antimicrobial efficacy.

Although the essential oils of *D. lanceolata* display promising antimicrobial properties, particularly against human pathogenic yeasts, several studies emphasize their potential application as adjuvants to commercially available antibiotics and antifungal drugs, including those targeting resistant microorganisms. Such combinations may reduce MIC values and mitigate adverse effects [65]. Essential oils combined with conventional antimicrobials can produce additive, antagonistic, indifferent, or synergistic outcomes. In synergistic systems, essential oils may exert multi‐target effects, enhance drug solubility and bioavailability, or interfere with specific resistance mechanisms [8]. Accordingly, further studies aimed at elucidating the mechanisms of action of *D. lanceolata* essential oils against *Candida* spp., *S. aureus*, and *A. baumannii* are warranted. Moreover, systematic evaluation of their activity in combination with established antimicrobial agents represents a crucial next step toward therapeutic application.

## 3 Conclusions

This study combined GC–MS analysis, molecular networking, in vitro antimicrobial assays, and in silico modeling to investigate the bioactivity of *D. lanceolata* essential oils. The oil obtained from leaves collected in autumn, characterized by a predominance of monoterpenes, exhibited significantly lower MIC values against *A. baumannii*, *C. krusei*, and *C. parapsilosis*, whereas the winter oil, enriched in sesquiterpenes, showed no statistically supported antimicrobial activity.

PASS prediction, molecular docking, and ADMET analyses consistently identified limonene as the main contributor to the observed biological activity. Rather than indicating stronger binding affinity alone, the in silico results revealed a more compact and efficient target engagement at essential microbial sites, combined with physicochemical properties compatible with favorable cellular accessibility and a low predicted risk of metabolic liabilities.

Overall, the statistically supported seasonal differences highlight the impact of environmental variation on essential oil composition and antimicrobial performance. These findings establish a rational basis for defining optimal harvesting periods and reinforce the value of integrating chemical profiling, biological evaluation, and in silico approaches to elucidate structure–activity relationships and guide the discovery of plant‐derived antimicrobial agents.

## 4 Experimental Section

### 4.1 Plant Material and Essential Oil Extraction

Leaves of *Duguetia lanceolata* St.‐Hil. (Annonaceae) were collected at a private farm (Gaspar Lopes Farm) located in the municipality of Alfenas, Minas Gerais State, Brazil (21°22′53.8″ S, 45°55′46.4″ W), within an Atlantic Forest biome area. Botanical identification was performed by Dr. Marcelo Polo, and a voucher specimen was prepared and deposited at the Herbarium of the Institute of Biosciences, University of São Paulo (IB‐USP), under the accession number J.P.C.03.2017.

Access to the Brazilian genetic heritage was duly registered in the National System for the Management of Genetic Heritage and Associated Traditional Knowledge (SisGen) under registration number A90708B, in compliance with Brazilian legislation.

Plant material was collected in 2022 during two distinct seasons in order to evaluate the influence of seasonal variation on essential oil composition and biological activity. Fresh leaves were collected in April (autumn) and July (winter). In each collection period, approximately 200 g of fresh leaves were used for essential oil extraction.

The essential oils were obtained by hydrodistillation using a modified Clevenger‐type apparatus, following the procedure described by Santos et al. [66]. Distillation was carried out for 5 h. After extraction, the oils were separated from the hydrolate using diethyl ether (Et_2_O), dried over anhydrous sodium sulfate (Na_2_SO_4_), filtered, and concentrated under reduced pressure. The essential oils obtained from autumn and winter collections were designated EO‐A and EO‐W, respectively. All samples were stored at −20°C until further chemical and biological analyses.

### 4.2 Gas Chromatography‐Mass Spectrometry (GC‐MS) Analysis

GC‐MS analyses were performed using a Shimadzu GC‐MS‐QP2010 system equipped with a DB‐5 capillary column (Agilent J&W; 30 m × 0.25 mm × 0.25 µm film thickness). The essential oils were diluted in diethyl ether (Et_2_O) to a concentration of 1.0 mg mL^−^^1^, and 1.0 µL was injected in split mode (1:10). Helium was used as the carrier gas. The injector and ion source temperatures were set at 220°C and 230°C, respectively. The oven temperature was programmed to start at 40°C, increasing at a rate of 3°C min^−^^1^ up to 240°C, which was maintained for 5 min. The MS operating conditions included an ionization voltage of 70 eV [67]. The chemical components were identified by comparing their mass spectra with those stored in the National Institute of Standards and Technology (NIST) library. Additionally, the identification was supported by comparing their linear retention indices (LRI) with literature data, calculated relative to a homologous series of *n*‐alkanes (C_8_–C_40_) analyzed under the same conditions. Quantitative determination was performed based on the peak area integration (normalization method) [67].

### 4.3 Untarget Metabolomic Assisted by GNPS—GC‐MS Spectral Library Search/Molecular Networking Workflow

The identification of compounds was performed through an untargeted metabolomic approach assisted by GNPS. Therefore, the experimental MS spectra obtained by GC‐MS analysis were compared with those available at the GNPS spectral library and with mass spectra available in literature, including the NIST 08 mass spectral library. A molecular network was created with the GC‐MS Spectral Library Search/Molecular Networking workflow at GNPS (https://gnps.ucsd.edu) [22, 23, 68]. Data were filtered by removing all MS fragment ions within ±17 Da of the precursor *m/z*. The MS spectra were window‐filtered by choosing only the top six fragment ions within a ±50 Da window throughout each spectrum. The precursor ion mass tolerance was set to 20 000 Da and the MS fragment ion tolerance to 0.65 Da. A molecular network was then created where edges were filtered to have a cosine score above 0.7 and more than 6 matched peaks. Finally, the maximum size of a molecular family was set to 100, and the lowest‐scoring edges were removed from molecular families until the molecular family size was below this threshold. The library spectra were filtered in the same manner as the input data [23]. All matches kept between network spectra and library spectra were required to have a score above 0.65 and at least 6 matched peaks [69]. The molecular networks were visualized using Cytoscape software version 3.10.3 [70].

### 4.4 Antimicrobial Activity Assays—Growth of Microorganisms in Solid Culture

The cultures were performed from the inoculum of the colonies isolated from the strains of interest in standard medium for YPD yeasts (1% yeast extract, 2% peptone, 2% dextrose, and 2% agar) and Luria–Bertani medium (Bio Basic Inc. S516), containing 25 mL of culture medium in each Petri dish (Table 2). Temperature and growth time ranged from 24 to 48 h and from 30°C to 37°C, depending on experimental planning. Eugenol and amphotericin‐B (200 µg mL^−1^) were used as positive controls for yeasts, and kanamycin and ampicillin (AppliChem) at 50 µg mL^−1^, as positive controls for bacteria. All susceptibility assays were performed in accordance with the guidelines established by the Clinical and Laboratory Standards Institute (CLSI) for disk diffusion and the European Committee on Antimicrobial Susceptibility Testing (EUCAST) [71, 72].

**TABLE 2:** Bacteria and yeast strains used for the disc diffusion method and minimum inhibition concentration assays.

Species	Lineage
Yeast strains	
**Cryptococcus neoformans**	KN99α (serotype A)
**Cryptococcus neoformans**	JEC21 (serotype D)
**Cryptococcus gattii**	NIH312 (serotype C)
**Cryptococcus gattii**	R265 (serotype B)
**Saccharomyces cerevisiae**	BY4647
**Candida krusei** ^a^	Clinical isolate 9602
**Candida parapsilosis**	Clinical isolate 68
**Candida albicans**	CBMAY 560
**Candida tropicalis**	ATCC 1303
**Candida dubliniensis**	ATCC 7876
**Bacteria**	
**Enterococcus faecium** ^b^	ATCC CCB076
**Pseudomonas aeruginosa** ^c^	ATCC 27853
**Klebsiella pneumoniae** ^d^	ATCC 700603
**Escherichia coli**	ATCC 25922
**Shigella flexneri**	ATCC 12022
**Salmonella enterica**	ATCC 14028
**Staphylococcus aureus**	ATCC25923
**Acinetobacter baumanii** ^e^	ATCC 19606

*Note*: Microorganisms associated with AMR.

^a^Clinical isolate with reduced susceptibility to caspofungin during therapy [73].

^b^Vancomycin‐resistant enterococci (VRE) are increasingly reported worldwide, with *E. faecium* exhibiting high resistance rates, particularly in South America and Iran [74].

^c^Strain resistant to carbapenems and polymyxins, with multiple antibiotic resistance mechanisms [75].

^d^Resistant to third‐ and fourth‐generation cephalosporins and KPC‐producing isolates, resistant to multiple β‐lactams, including carbapenems [76].

^e^Multidrug‐resistant strain, notably resistant to carbapenems, aminoglycosides, and quinolones [77].

### 4.5 Disk Diffusion Method

The disk diffusion method, to evaluate the antimicrobial susceptibility of microorganisms, was performed following the CLSI protocol (M2–A8) [71]. The microorganisms were cultured in LB or YPD medium 24 to 48 h before. The inoculum was made from the removal of 3 colonies and transferred to a test tube containing 5 mL of 0.9% NaCl saline solution. The suspension was adjusted to a cell density similar to the 0.5 McFarland scale (corresponds to 1.5 × 10^8^ cells per mL). Subsequently, with a sterile swab, the solution was spread evenly over the plate containing the Mueller Hinton medium (BD 212322) for bacteria or the Mueller Hinton medium supplemented with D‐glucose (2%) and methylene blue (0.5 µg mL^−1^) for yeasts. Then, 5 mm diameter sterile filter paper discs (Whatman #1), which received 5 to 10 µL of tested essential oils with a final concentration range of 400 µg mL^−1^, were laid on top of the culture medium. Positive controls containing filter paper discs were also placed (air‐dried discs impregnated with 200 µg of eugenol and amphotericin‐B for yeasts or 50 µg of kanamycin for bacteria) and a negative control (air‐dried discs impregnated with DMSO). The plates were incubated at 37°C for 24 h (bacteria) and at 30°C for 48 h (yeast), and, after this period, the halos were measured using a pachymeter and the data duly recorded. Results express the data collected from three distinct biological replicates.

### 4.6 MIC

Microdilution tests in broth were performed in a 96‐well plate for bacteria, using the EUCAST (European Antimicrobial Susceptibility Testing Committee) method [72, 78]. Briefly, bacteria (Table 2) were grown in solid LB medium for 24 h at 37°C, while yeasts (Table 2) were grown in solid YPD medium for 48 h at 30°C. Three fresh colonies were transferred to 5 mL of saline solution (NaCl at 0.9%); this cell suspension was adjusted for a cell density of approximately 1–2 × 10^8^ × CFU/mL (0.5 McFarland scale).

The tested essential oils were diluted in DMSO and adjusted to an initial concentration of 0.541 µg mL^−1^. Microtiter plates with 96 wells and a total volume of 100.0 µL were used to calculate the MIC values. The cultures were diluted and adjusted to 1–2 × 10^2^ CFU/mL using a McFarland scale. The compounds were serially diluted (two‐fold) and added to each well. A negative control, containing medium only, and a growth control (positive control), containing cells, DMSO (10.0 µL), and saline (10.0 µL), were included. Depending on the microorganism (bacteria or yeast), the microtiter plates were then incubated at 37°C for 24 h or 30°C for 48 h. Microorganism growth was determined by reading the absorbance at 530 nm in a plate reader (Logen, MT‐960, BioTek Instruments, Winooski, VT, USA), and the minimum inhibitory concentration was considered the lowest concentration at which at least 90% of growth was inhibited. All tests were performed in triplicate.

### 4.7 Statistical Analysis—ANOVA

All statistical analyses were performed using jamovi software (version 2.6.44; https://www.jamovi.org) [79, 80]. Experimental data were expressed as mean ± standard deviation (SD) or standard error (SE), as appropriate. Differences among treatments were evaluated using one‐way analysis of variance (ANOVA). When assumptions of homogeneity of variances were not met, one‐way ANOVA (Welch's) was applied. Post hoc comparisons were conducted using Tukey's or Games–Howell tests, as appropriate. Differences were considered statistically significant at *p* < 0.05 [81, 82].

### 4.8 In Silico Analysis

All in silico analyses were performed to support and rationalize the experimental antimicrobial data obtained for the essential oils and their major constituents.

***PASS Prediction of Biological Activity (PASS Online)—*** The Prediction of Activity Spectra for Substances (https://way2drug.com/PassOnline/) platform was employed to estimate the probability of antibacterial and antifungal activities of the major terpenes. Canonical SMILES strings of limonene and β‐bisabolene were used as input. PASS provides probabilities to be active (Pa) and inactive (Pi) based on structure–activity relationships derived from a large curated database. Compounds presenting Pa values between 0.3 and 0.7 were considered to exhibit moderate but biologically relevant activity profiles [83].

***Molecular Docking—*** Simulations were carried out using SwissDock (http://www.swissdock.ch), which is based on the EADock DSS engine and the Attracting Cavities 2.0 algorithm [53, 54]. Two biologically relevant targets were selected: DNA gyrase B from *A. baumannii* (PDB: 7PQL; x‐ray diffraction), as a representative antibacterial target. Chain A (DNA gyrase subunit B) was retained, along with heteroatom 80S A 301 [52, 58]. Lanosterol 14α‐demethylase (CYP51) from *C. albicans* (PDB: 5FSA; x‐ray diffraction) as a model antifungal target. Chain A (lanosterol 14‐alpha demethylase) was retained, along with heteroatom HEM A 580 [84, 85]. The ligands limonene (CC1=CCC(CC1)C(=C)C) and β‐bisabolene (CC1=CCC@HC(=C)CCC=C(C)C) were submitted in SMILES format and automatically prepared by the server [86].

Docking calculations were performed using the Attracting Cavities 2.0 protocol with medium sampling exhaustivity and one region of interest cluster (RIC = 1). For DNA gyrase, the search space was centered at coordinates (205, −14, −58), and for CYP51 at (−8, −14, 15), using a cubic box of 20 × 20 × 20 Å in both cases. Cavities were prioritized as “buried,” targeting catalytically relevant pockets. For each system, the best‐ranked binding pose of the top cluster was selected based on the predicted binding free energy (Δ*G*, kcal mol^−^^1^). Docking poses and interaction maps were visualized and analyzed using Discovery Studio Visualizer (version 21.1.0) [51].

***ADME/T Prediction—*** Pharmacokinetic and toxicity‐related properties were predicted using the SwissADME platform (https://www.swissadme.ch) [87]. The analysis included physicochemical descriptors, Lipinski's Rule of Five, solubility estimates (ESOL and Ali models), bioavailability score, gastrointestinal absorption, blood–brain barrier permeation (Boiled‐Egg model), and interaction probabilities with major human cytochrome P450 isoforms (CYP1A2, CYP2C9, CYP2C19, CYP2D6, and CYP3A4). These parameters were used to evaluate the translational potential and metabolic safety of limonene and β‐bisabolene in the context of their antimicrobial activity [59, 61, 87].

## Author Contributions

**Jackson Monteiro**: conceptualization, methodology, software, formal analysis, review and editing. **Geovanna N. Antonelli**: methodology, formal analysis. **Erick P. Dantas**: methodology. **Simone S. Grecco**: conceptualization, methodology, software, formal analysis, preparation of original draft, review and editing. **Marisi G. Soares**: data curation**. João Henrique G. Lago**: review and editing. **Renata C. Pascon**: methodology, formal analysis, data curation, review and editing, acquisition of funding. **Marcelo A. Vallim**: methodology, formal analysis, data curation, review and editing, supervision, acquisition of funding. **Patricia Sartorelli**: conceptualization, formal analysis, resources, data curation, preparation of original draft, review and editing, supervision, project administration, acquisition of funding. All authors have read and agreed to the published version of the manuscript.

## Conflicts of Interest

The authors declare no conflicts of interest.

## Supporting information




**Supporting File 1**: cbdv70998‐sup‐0001‐SuppMat.docx

**Supporting File 2**: cbdv70998‐sup‐0002‐FigureS1.TIF

**Supporting File 3**: cbdv70998‐sup‐0003‐FigureS2.TIF

**Supporting File 4**: cbdv70998‐sup‐0004‐FigureS3.TIF

**Supporting File 5**: cbdv70998‐sup‐0005‐FigureS4.TIF

**Supporting File 6**: cbdv70998‐sup‐0006‐FigureS5.tif

## Data Availability

The authors have nothing to report.

## References

[1] L. Morrison and T. R. Zembower , “Antimicrobial Resistance,” Gastrointestinal Endoscopy Clinics of North America 30 (2020): 619–635, 10.1016/j.giec.2020.06.004. 32891221

[2] World Health Organization , Antimicrobial‐Resistance (WHO, 2023), accessed September 18, https://www.who.int/news‐room/fact‐sheets/detail/antimicrobial‐resistance.

[3] J. Denissen , B. Reyneke , M. Waso‐Reyneke , et al., “Prevalence of ESKAPE Pathogens in the Environment: Antibiotic Resistance Status, Community‐Acquired Infection and Risk to Human Health,” International Journal of Hygiene and Environmental Health 244 (2022): 114006, 10.1016/j.ijheh.2022.114006. 35841823

[4] D. J. Newman and G. M. Cragg , “Natural Products as Sources of New Drugs Over the Nearly Four Decades From 01/1981 to 09/2019,” Journal of Natural Products 83 (2020): 770–803, 10.1021/acs.jnatprod.9b01285. 32162523

[5] F. J. Álvarez‐Martínez , R. Díaz‐Puertas , E. Barrajón‐Catalán , et al., “Plant‐Derived Natural Products for the Treatment of Bacterial Infections,” in Handbook of Experimental Pharmacology, ed. M. M. Michel (Springer, 2025), 10.1007/164_2024_706. 38418668

[6] A. I. Visan and I. Negut , “Coatings Based on Essential Oils for Combating Antibiotic Resistance,” Antibiotics 13 (2024): 625, 10.3390/antibiotics13070625. 39061307 PMC11273621

[7] R. Iseppi , M. Mariani , C. Condò , C. Sabia , and P. Messi , “Essential Oils: A Natural Weapon Against Antibiotic‐Resistant Bacteria Responsible for Nosocomial Infections,” Antibiotics 10 (2021): 417, 10.3390/antibiotics10040417. 33920237 PMC8070240

[8] G. Raikwar , D. Kumar , S. Mohan , and P. Dahiya , “Synergistic Potential of Essential Oils With Antibiotics for Antimicrobial Resistance With Emphasis on Mechanism of Action: A Review,” Biocatalysis and Agricultural Biotechnology 61 (2024): 103384, 10.1016/j.bcab.2024.103384.

[9] S.‐K. Yang , N.‐P. Tan , C.‐W. Chong , A. Abushelaibi , S.‐H.‐E. Lim , and K.‐S. Lai , “The Missing Piece: Recent Approaches Investigating the Antimicrobial Mode of Action of Essential Oils,” Evolutionary Bioinformatics 17 (2021): 1176934320938391, 10.1177/1176934320938391. 34017165 PMC8114247

[10] K. A. El‐Tarabily , M. T. El‐Saadony , M. Alagawany , et al., “Using Essential Oils to Overcome Bacterial Biofilm Formation and Their Antimicrobial Resistance,” Saudi Journal of Biological Sciences 28 (2021): 5145–5156, 10.1016/j.sjbs.2021.05.033. 34466092 PMC8380992

[11] J. C. E. de Queiroz , J. R. S. A. Leite , and A. G. Vasconcelos , “Prospecting Plant Extracts and Bioactive Molecules With Antimicrobial Activity in Brazilian Biomes: A Review,” Antibiotics 12 (2023): 427, 10.3390/antibiotics12030427. 36978294 PMC10044579

[12] O. O. Ferreira , J. N. Cruz , Â. A. B. De Moraes , et al., “Essential Oil of the Plants Growing in the Brazilian Amazon: Chemical Composition, Antioxidants, and Biological Applications,” Molecules (Basel, Switzerland) 27 (2022): 4373, 10.3390/molecules27144373. 35889245 PMC9318482

[13] F. Médail and N. Myers , “Mediterranean Basin,” in Hotspots Revisited: Earth's Biologically Richest and Most Endangered Terrestrial Ecoregions, eds. R. A. Mittermeier , P. Robles‐Gil , M. Hoffmann , et al., (CEMEX, Conservation International, and Agrupación Sierra Madre, 2004), 144–147.

[14] J. R. G. S. Almeida , J. T. De Lima , H. R. De Oliveira , et al., “Antinociceptive Activity of Discretamine Isolated From *Duguetia moricandiana* ,” Natural Product Research 25 (2011): 1908–1915, 10.1080/14786419.2010.491227. 21656417

[15] I. Isernhagen , Listagem Florística de Espécies Arbóreas e Arbustivas de Mato Grosso: um Ponto de Partida Para Projetos de Restauração Ecológica. 4th ed. (Embrapa Agrossilvipastoril, 2015).

[16] A. Q. Lobão and M. L. Bazante , Duguetia (Flora e Funga Do Brasil, 2025).

[17] D. C. H. Fischer , N. C. De Amorim Gualda , D. Bachiega , et al., “In Vitro Screening for Antiplasmodial Activity of Isoquinoline Alkaloids From Brazilian Plant Species,” Acta Tropica 92 (2004): 261–266, 10.1016/j.actatropica.2004.08.009. 15533296

[18] O. V. Sousa , G. Del‐Vechio‐Vieira , M. P. H. Amaral , et al., “Efeitos Antinociceptivo E Antiinflamatório do Extrato Etanólico Das Folhas de *Duguetia lanceolata* St.‐Hil. (Annonaceae),” Latin American Journal of Pharmacy 27 (2008): 398–402.

[19] O. V. Sousa , D. T. Soares Júnior , G. Del‐Vechio , R. G. Mattosinhos , C. R. Gattass , and M. A. C. Kaplan , “Atividades Antinociceptiva e Antiinflamatória Do Óleo Essencial de Cascas De Duguetia lanceolata St. Hil., Annonaceae,” Hil, Annonaceae Revista Brasileira de Farmacognosia 14 (2004): 11–14, 10.1590/S0102-695X2004000300005.

[20] O. V. Sousa , G. Del‐Vechio‐Vieira , M. S. Alves , et al., “Chemical Composition and Biological Activities of the Essential Oils From *Duguetia lanceolata* St. Hil. Barks,” Hil Barks Molecules 17 (2012): 11056–11066, 10.3390/molecules170911056. 22976469 PMC6268367

[21] L. P. Ribeiro , V. C. Domingues , G. L. P. Gonçalves , J. B. Fernandes , E. M. Glória , and J. D. Vendramim , “Essential Oil From *Duguetia lanceolata* St.‐Hil. (Annonaceae): Suppression of Spoilers of Stored‐Grain,” Food Bioscience 36 (2020): 100653, 10.1016/j.fbio.2020.100653.

[22] A. A. Aksenov , I. Laponogov , Z. Zhang , et al., “Auto‐Deconvolution and Molecular Networking of Gas Chromatography–Mass Spectrometry Data,” Nature Biotechnology 39 (2021): 169–173, 10.1038/s41587-020-0700-3. PMC7971188 33169034

[23] M. Wang , J. J. Carver , V. V. Phelan , et al., “Sharing and Community Curation of Mass Spectrometry Data With Global Natural Products Social Molecular Networking,” Nature Biotechnology 34 (2016): 828–837, 10.1038/nbt.3597. PMC5321674 27504778

[24] M. M. Cascaes , O. Dos Santos Carneiro , L. D. Do Nascimento , et al., “Essential Oils From Annonaceae Species From Brazil: A Systematic Review of Their Phytochemistry, and Biological Activities,” International Journal of Molecular Sciences 22 (2021): 12140, 10.3390/ijms222212140. 34830022 PMC8623146

[25] J. M. Alcântara , J. M. V. M. De Lucena , R. Facanali , et al., “Chemical Composition and Bactericidal Activity of the Essential Oils of Four Species of Annonaceae Growing in Brazilian Amazon,” Natural Products Communications 12 (2017): 619–622, 10.1177/1934578X1701200437. 30520609

[26] A. C. D. Santos , M. L. Nogueira , and F. P. de Oliveira , E. V. Costa , and D. P. Bezerra , “Essential Oils of *Duguetia* Species A. St. Hill (Annonaceae): Chemical Diversity and Pharmacological Potential.” Biomolecules 12 (2022): 615, 10.3390/biom12050615. 35625543 PMC9138787

[27] G. Fournier , A. Hadjiakhoondi , M. Leboeuf , A. Cavé , and B. Charles , “Essential Oils of Annonaceae. Part VIII. Volatile Constituents of the Essential Oils From Three *Guatteria* Species,” Journal of Essential Oil Research 9 (1997): 275–278, 10.1080/10412905.1997.10554243.

[28] J. G. S. Maia , E. H. A. Andrade , A. C. M. Da Silva , J. Oliveira , L. M. M. Carreira , and J. S. Araújo , “Leaf Volatile Oils From Four Brazilian *Xylopia* Species,” Flavour and Fragrance Journal 20 (2005): 474–477, 10.1002/ffj.1499.

[29] J. G. S. Maia , E. H. A. Andrade , L. M. M. Carreira , J. Oliveira , and J. S. Araújo , “Essential Oils of the Amazon *Guatteria* and *Guatteriopsis* Species,” Flavour and Fragrance Journal 20 (2005): 478–480, 10.1002/ffj.1500.

[30] C. Zidorn , “Plant Chemophenetics − A New Term for Plant Chemosystematics/Plant Chemotaxonomy in the Macro‐Molecular Era,” Phytochemistry 163 (2019): 147–148, 10.1016/j.phytochem.2019.02.013. 30846237

[31] V. De Sousa Orlando , D. V. V. Glauciemar , C. S. S. Bruna , et al., “In‐ Vivo and Vitro Bioactivities of the Essential Oil of *Duguetia lanceolata* Branches,” African Journal of Pharmacy and Pharmacology 10 (2016): 298–310, 10.5897/AJPP2015.4497.

[32] D. S. Maia , C. F. Lopes , A. A. Saldanha , et al., “Larvicidal Effect From Different Annonaceae Species on *Culex quinquefasciatus* ,” Environmental Science and Pollution Research 27 (2020): 36983–36993, 10.1007/s11356-020-08997-6. 32577964

[33] A. Laftouhi , N. Eloutassi , E. Ech‐Chihbi , et al., “The Impact of Environmental Stress on the Secondary Metabolites and the Chemical Compositions of the Essential Oils From Some Medicinal Plants Used as Food Supplements,” Sustainability 15 (2023): 7842, 10.3390/su15107842.

[34] C. M. Germano , N. R. Ruas , O. A. Lameira , et al., “Seasonal Variations During Two Years in the Essential Oil of *Lippia dulcis* Trevir., An Exotic Aromatic of the Amazon,” Journal of Essential Oil Research 34 (2022): 352–360, 10.1080/10412905.2022.2058635.

[35] A. Punetha , D. Kumar , P. Suryavanshi , R. C. Padalia , and V. K. Thimmaiah , “Environmental Abiotic Stress and Secondary Metabolites Production in Medicinal Plants: A Review,” Journal of Agricultural Sciences (Tarım Bilimleri Dergisi) 28 (2022): 351–362, 10.15832/ankutbd.999117.

[36] A. Sharma , A. Biharee , A. Kumar , and V. Jaitak , “Antimicrobial Terpenoids as a Potential Substitute in Overcoming Antimicrobial Resistance,” Current Drug Targets 21 (2020): 1476–1494, 10.2174/1389450121666200520103427. 32433003

[37] T. Ganić , S. Vuletić , B. Nikolić , et al., “Cinnamon Essential Oil and Its Emulsion as Efficient Antibiofilm Agents to Combat *Acinetobacter baumannii* ,” Frontiers in Microbiology 13 (2022): 989667, 10.3389/fmicb.2022.989667. 36299724 PMC9589355

[38] K. Hao , B. Xu , G. Zhang , et al., “Antibacterial Activity and Mechanism of *Litsea Cubeba* L. Essential Oil against *Acinetobacter baumannii* ,” Natural Products Communications 16 (2021): 1934578X2199914, 10.1177/1934578X21999146.

[39] A. I. Jaisankar , A. S. S. Girija , S. Gunasekaran , and J. V. Priyadharsini , “Molecular Characterisation of csgA Gene Among ESBL Strains of *A. baumannii* and Targeting With Essential Oil Compounds From *Azadirachta indica* ,” Journal of King Saud University ‐ Science 32 (2020): 3380–3387, 10.1016/j.jksus.2020.09.025.

[40] M. H. Pereira De Lira , G. F. Q. Moraes , G. Macena Santos , F. P. De Andrade Júnior , F. De Oliveira Pereira , and I. O. Lima , “Synergistic Antibacterial Activity of Monoterpenes in Combination With Conventional Antimicrobials Against *Gram*‐Positive and *Gram*‐Negative Bacteria,” Revista de Ciências Médicas e Biológicas 19 (2020): 258, 10.9771/cmbio.v19i2.33665.

[41] K. J. S. De Oliveira Dias , G. M. Miranda , J. R. Bessa , et al., “Terpenes as Bacterial Efflux Pump Inhibitors: A Systematic Review,” Frontiers in Pharmacology 13 (2022): 953982, 10.3389/fphar.2022.953982. 36313340 PMC9606600

[42] J. Sikkema , J. A. M. de Bont , and B. Poolman , “Interactions of Cyclic Hydrocarbons With Biological Membranes,” Journal of Biological Chemistry 269 (1997): 8022–8028, 10.1016/S0021-9258(17)37154-5. 8132524

[43] M. Cristani , M. D'Arrigo , G. Mandalari , et al., “Interaction of Four Monoterpenes Contained in Essential Oils With Model Membranes: Implications for Their Antibacterial Activity,” Journal of Agricultural and Food Chemistry 55 (2007): 6300–6308, 10.1021/jf070094x. 17602646

[44] C. M. O. Simões , E. P. Schenkel , J. C. P. de Mello , et al., Farmacognosia: Do Produto Natural Ao Medicamento. 1st ed. (Artmeda, 2016).

[45] P. Noriega , J. Ballesteros , A. De La Cruz , and T. Veloz , “Chemical Composition and Preliminary Antimicrobial Activity of the Hydroxylated Sesquiterpenes in the Essential Oil From *Piper barbatum* Kunth Leaves,” Plants 9 (2020): 211, 10.3390/plants9020211. 32041311 PMC7076699

[46] A. L. Ogundajo , T. Ewekeye , O. J. Sharaibi , M. S. Owolabi , N. S. Dosoky , and W. N. Setzer , “Antimicrobial Activities of Sesquiterpene‐Rich Essential Oils of Two Medicinal Plants, *Lannea egregia* and *Emilia sonchifolia*, From Nigeria,” Plants 10 (2021): 488, 10.3390/plants10030488. 33807551 PMC8000775

[47] A. Lagunin , A. Stepanchikova , D. Filimonov , and V. Poroikov , “PASS: Prediction of Activity Spectra for Biologically Active Substances,” Bioinformatics 16 (2000): 747–748, 10.1093/bioinformatics/16.8.747. 11099264

[48] R. C. E Silva , J. S. Da Costa , and R. O. De Figueiredo , et al., “Monoterpenes and Sesquiterpenes of Essential Oils From *Psidium* Species and Their Biological Properties,” Molecules (Basel, Switzerland) 26 (2021): 965, 10.3390/molecules26040965. 33673039 PMC7917929

[49] M. Kara , N. Haoudi , N. El Houda Tahiri , et al., “Chemical Profiling, Antioxidant and Antimicrobial Activities, and *in Silico* Evaluation of *Gardenia jasminoides* Essential Oil,” Plants 14 (2025): 1055, 10.3390/plants14071055. 40219122 PMC11991191

[50] M. S. Bahia , O. Kaspi , M. Touitou , et al., “A Comparison Between 2D and 3D Descriptors in QSAR Modeling Based on Bio‐Active Conformations,” Molecular Informatics 42 (2023): e2200186, 10.1002/minf.202200186. 36617991

[51] A. M. Fahim , “Structure‐Based Drug Design; Computational Strategies in Drug Discovery; Antihypertensive Agents; Antiviral Drugs; Molecular Docking; QSAR; Pharmacological Insights,” Computational Biology and Chemistry 120 (2026): 108663, 10.1016/j.compbiolchem.2025.108663. 40914997

[52] G. Zhou and Y. Li , “Investigation of Bacterial DNA Gyrase Inhibitor Classification Models and Structural Requirements Utilizing Multiple Machine Learning Methods,” Molecular Diversity 28 (2024): 2119–2133, 10.1007/s11030-024-10806-y. 38372837

[53] U. F. Röhrig , M. Goullieux , M. Bugnon , et al., “Attracting Cavities 2.0: Improving the Flexibility and Robustness for Small‐Molecule Docking,” Journal of Chemical Information and Modeling 63 (2023): 3925–3940, 10.1021/acs.jcim.3c00054. 37285197 PMC10305763

[54] M. Bugnon , U. F. Röhrig , M. Goullieux , et al., “SwissDock 2024: Major Enhancements for Small‐Molecule Docking With Attracting Cavities and AutoDock Vina,” Nucleic Acids Research 52 (2024): W324–W332, 10.1093/nar/gkae300. 38686803 PMC11223881

[55] T. V. Tsybruk , L. A. Kaluzhskiy , Y. V. Mezentsev , et al., “Molecular Cloning, Heterologous Expression, Purification, and Evaluation of Protein–Ligand Interactions of CYP51 of *Candida krusei* Azole‐Resistant Fungal Strain,” Biomedicines 11 (2023): 2873, 10.3390/biomedicines11112873. 38001874 PMC10668980

[56] A. Gupta , E. Jeyakumar , and R. Lawrence , “Strategic Approach of Multifaceted Antibacterial Mechanism of Limonene Traced in *Escherichia coli* ,” Scientific Reports 11 (2021): 13816, 10.1038/s41598-021-92843-3. 34226573 PMC8257740

[57] J. O. E. Nogueira , G. A. Campolina , L. R. Batista , et al., “Mechanism of Action of Various Terpenes and Phenylpropanoids Against *Escherichia coli* and *Staphylococcus aureus* ,” FEMS Microbiology Letters 368 (2021): fnab052, 10.1093/femsle/fnab052. 34003259

[58] M. T. Muhammed and E. Aki‐Yalcin , “Computational Insight Into the Mechanism of Action of DNA Gyrase Inhibitors; Revealing a New Mechanism,” Current Computer‐Aided Drug Design 20 (2023): 224–235, 10.2174/1573409919666230419094700. 37114781

[59] R. Ancuceanu , B. E. Lascu , D. Drăgănescu , and M. Dinu , “In Silico ADME Methods Used in the Evaluation of Natural Products,” Pharmaceutics 17 (2025): 1002, 10.3390/pharmaceutics17081002. 40871023 PMC12389637

[60] A. E. Edris , “Pharmaceutical and Therapeutic Potentials of Essential Oils and Their Individual Volatile Constituents: A Review,” Phytotherapy Research 21 (2007): 308–323, 10.1002/ptr.2072. 17199238

[61] A. Daina and V. Zoete , “A BOILED‐Egg To Predict Gastrointestinal Absorption and Brain Penetration of Small Molecules,” ChemMedChem 11 (2016): 1117–1121, 10.1002/cmdc.201600182. 27218427 PMC5089604

[62] T. Siddiqui , M. U. Khan , V. Sharma , and K. Gupta , “Terpenoids in Essential Oils: Chemistry, Classification, and Potential Impact on human Health and Industry,” Phytomedicine Plus 4 (2024): 100549, 10.1016/j.phyplu.2024.100549.

[63] J. M. M. Araujo , J. M. Monteiro , D. H. Dos Santos Silva , et al., “ *Candida krusei* M4CK Produces a Bioemulsifier That Acts on Melaleuca Essential Oil and Aids in Its Antibacterial and Antibiofilm Activity,” Antibiotics 12 (2023): 1686, 10.3390/antibiotics12121686. 38136720 PMC10740703

[64] M. H. G. Novais , N. S. Farias , A. G. Dos Santos , et al., “Pharmacological Potential of Limonene Against Opportunistic Fungi: Impact on *Candida* Virulence,” Acta Tropica 253 (2024): 107168, 10.1016/j.actatropica.2024.107168. 38432404

[65] L. Dos Santos Janotto , T. De Melo Nazareth , G. Meca , F. B. Luciano , and A. G. Evangelista , “Exploring the Efficacy of Antibiotic‐Essential Oil Combinations: Implications for Combating Antimicrobial Resistance,” Bioresource Technology Reports 24 (2023): 101679, 10.1016/j.biteb.2023.101679.

[66] N. Santos , R. Pascon , M. Vallim , et al., “Cytotoxic and Antimicrobial Constituents From the Essential Oil of *Lippia alba* (Verbenaceae),” Medicines 3 (2016): 22, 10.3390/medicines3030022. 28930132 PMC5456251

[67] R. P. Adams , Identification of Essential Oil Components by Gas Chromatograpy/Mass Spectrometry. 4th ed. (Allured Publishing Corporation, 2007), 804–806.

[68] G. S. Dos Santos , M. V. Sousa Teixeira , L. Da Costa Clementino , et al., “Annotation of GC‐MS Data of Antimicrobial Constituents in the Antarctic Seaweed Phaeurus Antarcticus by Molecular Networking,” Chemistry and Biodiversity 20 (2023): e202300429, 10.1002/cbdv.202300429. 37908056

[69] H. Mohimani , A. Gurevich , A. Shlemov , et al., “Dereplication of Microbial Metabolites Through Database Search of Mass Spectra,” Nature Communications 9 (2018): 4035, 10.1038/s41467-018-06082-8. PMC6168521 30279420

[70] D. Otasek , J. H. Morris , J. Bouças , A. R. Pico , and B. Demchak , “Cytoscape Automation: Empowering Workflow‐Based Network Analysis,” Genome Biology 20 (2019): 185, 10.1186/s13059-019-1758-4. 31477170 PMC6717989

[71] M. Weinstein , CLSI M100 Performance Standards for Antimicrobial Susceptibility Testing (CLSI, 2021).

[72] E. Matuschek , S. Copsey‐Mawer , S. Petersson , J. Åhman , T. E. Morris , and G. Kahlmeter , “The European Committee on Antimicrobial Susceptibility Testing Disc Diffusion Susceptibility Testing Method for Frequently Isolated Anaerobic Bacteria,” Clinical Microbiology and Infection 29 (2023). 795.e1–795.e7, 10.1016/j.cmi.2023.01.027. 36746258

[73] M. Hakki , J. F. Staab , and K. A. Marr , “Emergence of a *Candida krusei* Isolate With Reduced Susceptibility to Caspofungin During Therapy,” Antimicrobial Agents and Chemotherapy 50 (2006): 2522–2524, 10.1128/AAC.00148-06. 16801435 PMC1489805

[74] F. Menichetti , “Current and Emerging Serious *Gram*‐Positive Infections,” Clinical Microbiology and Infection 11 (2005): 22–28, 10.1111/j.1469-0691.2005.01138.x. 15811021

[75] J.‐I. Sekiguchi , T. Asagi , T. Miyoshi‐Akiyama , et al., “Multidrug‐Resistant *Pseudomonas aeruginosa* Strain That Caused an Outbreak in a Neurosurgery Ward and Its *Aac(6′)‐Iae* Gene Cassette Encoding a Novel Aminoglycoside Acetyltransferase,” Antimicrobial Agents and Chemotherapy 49 (2005): 3734–3742, 10.1128/AAC.49.9.3734-3742.2005. 16127047 PMC1195402

[76] J. K. Rasheed , G. J. Anderson , H. Yigit , et al., “Characterization of the Extended‐Spectrum β‐Lactamase Reference Strain, *Klebsiella pneumoniae* K6 (ATCC 700603), Which Produces the Novel Enzyme SHV‐18,” Antimicrobial Agents and Chemotherapy 44 (2000): 2382–2388, 10.1128/AAC.44.9.2382-2388.2000. 10952583 PMC90073

[77] M. Hamidian , L. Blasco , L. N. Tillman , J. To , M. Tomas , and G. S. A. Myers , “Analysis of Complete Genome Sequence of *Acinetobacter baumannii* Strain ATCC 19606 Reveals Novel Mobile Genetic Elements and Novel Prophage,” Microorganisms 8 (2000): 1851, 10.3390/microorganisms8121851. PMC7760358 33255319

[78] G. Kahlmeter , D. F. J. Brown , F. W. Goldstein , et al., “European Committee on Antimicrobial Susceptibility Testing (EUCAST) Technical Notes on Antimicrobial Susceptibility Testing,” Clinical Microbiology and Infection 12 (2006): 501–503, 10.1111/j.1469-0691.2006.01454.x. 16700696

[79] N. A. Milanes‐Baños , “Step‐by‐Step One‐Way ANOVA Analysis With the Jamovi Program,” Mexican Journal of Medical Research ICSA 12 (2024): 22–26, 10.29057/mjmr.v12i23.10664.

[80] Jamovi , The jamovi project, jamovi (Version 2.5) [Computer Software], 2024, https://www.jamovi.org.

[81] P. A. Games and J. F. Howell , “Pairwise Multiple Comparison Procedures With Unequal N's and/or Variances: A Monte Carlo Study,” Journal of Educational Statistics 1 (1976): 113–125, 10.3102/10769986001002113.

[82] B. L. Welch , “On the Comparison of Several Mean Values: An Alternative Approach,” Biometrika 38 (1951): 330–336, 10.2307/2332579.

[83] D. A. Filimonov , A. A. Lagunin , T. A. Gloriozova , et al., “Prediction of the Biological Activity Spectra of Organic Compounds Using the Pass Online Web Resource,” Chemistry of Heterocyclic Compounds (N Y) 50 (2014): 444–457, 10.1007/s10593-014-1496-1.

[84] J. Zhang , L. Li , Q. Lv , et al., “The Fungal CYP51s: Their Functions, Structures, Related Drug Resistance, and Inhibitors,” Frontiers in Microbiology 10 (2019): 691, 10.3389/fmicb.2019.00691. 31068906 PMC6491756

[85] A. G. S. Warrilow , C. M. Martel , J. E. Parker , et al., “Azole Binding Properties of *Candida albicans* Sterol 14‐α Demethylase (CaCYP51),” Antimicrobial Agents and Chemotherapy 54 (2010): 4235–4245, 10.1128/AAC.00587-10. 20625155 PMC2944560

[86] S. Kim , P. A. Thiessen , E. E. Bolton , et al., “PubChem Substance and Compound Databases,” Nucleic Acids Research 44 (2016): D1202–D1213, 10.1093/nar/gkv951. 26400175 PMC4702940

[87] A. Daina , O. Michielin , and V. Zoete , “SwissADME: A Free Web Tool to Evaluate Pharmacokinetics, Drug‐likeness and Medicinal Chemistry Friendliness of Small Molecules,” Scientific Reports 7 (2017): 42717, 10.1038/srep42717. 28256516 PMC5335600

