# Supplementary material for: GNPS Untargeted GC‐MS Metabolomic Analysis of Essential Oils From Duguetia lanceolata and Evaluation of Antimicrobial Activity
Source: Chem Biodivers. 2026 Feb 25;23(2):e03280. doi: 10.1002/cbdv.202503280 (PMC12935287; doi:10.1002/cbdv.202503280)
Supplement: Supplementary file 1 — Supporting File 1: cbdv70998‐sup‐0001‐SuppMat.docx [file CBDV-23-e03280-s003.docx]

Supplementary Material

***Figure S1.*** *GC-MS total ion chromatograms of essential oils from D. lanceolata leaves. (EO-A and EO-W)....................................................2****Figure S2.*** *In silico ADME/T profile of limonene predicted by SwissADME, including physicochemical properties, drug-likeness filters, lipophilicity, solubility, and cytochrome P450 inhibition probabilities……………………………………………………………………………….....2****Figure S3.*** *In silico ADME/T profile of β-bisabolene predicted by SwissADME, including physicochemical properties, drug-likeness filters, lipophilicity, solubility, and cytochrome P450 interaction probabilities………………………………………………………………………….….…3****Figure S4.*** *“Boiled-Egg” model illustrating the predicted gastrointestinal absorption and blood–brain barrier (BBB) permeation of limonene and β-bisabolene. The yolk (yellow) represents the region of high BBB permeation probability, while the white region corresponds to high intestinal absorption. The positions of the two terpenes highlight distinct distribution profiles, with limonene located within the BBB-permeant region and β-bisabolene positioned outside this zone, indicating restricted central nervous system access……………………………………..3****Figure S6.*** *CYPlebrity radar plot showing the predicted interaction of limonene and β-bisabolene with major human cytochrome P450 isoforms, indicating a generally low to moderate inhibition potential and a favorable metabolic safety profile................................................3*

[***Table S1.*** *Chemical composition of the essential oil of D. lanceolata (EO-A and EO-W) - List of annotated compounds by GNPS. 4*](#_Toc216961300)

[***Table S2.*** *Statistical analysis – ANOVA: Disk Diffusion Method - Zone of inibition (cm) 7*](#_Toc216961301)

[***Table S3.*** *One-Way ANOVA (Welch's) 8*](#_Toc216961302)

[***Table S4****. Homogeneity of Variances Test (Levene's) 8*](#_Toc216961303)

[***Table S5.*** *Post Hoc Tests: Tukey Post-Hoc Test 8*](#_Toc216961304)

[***Table S6.*** *Statistical analysis – ANOVA: MIC_90_ 10*](#_Toc216961305)

[***Table S7.*** *One-Way ANOVA (Welch's) 10*](#_Toc216961306)

[***Table S8.*** *Post Hoc Tests: Tukey Post-Hoc Test 11*](#_Toc216961307)


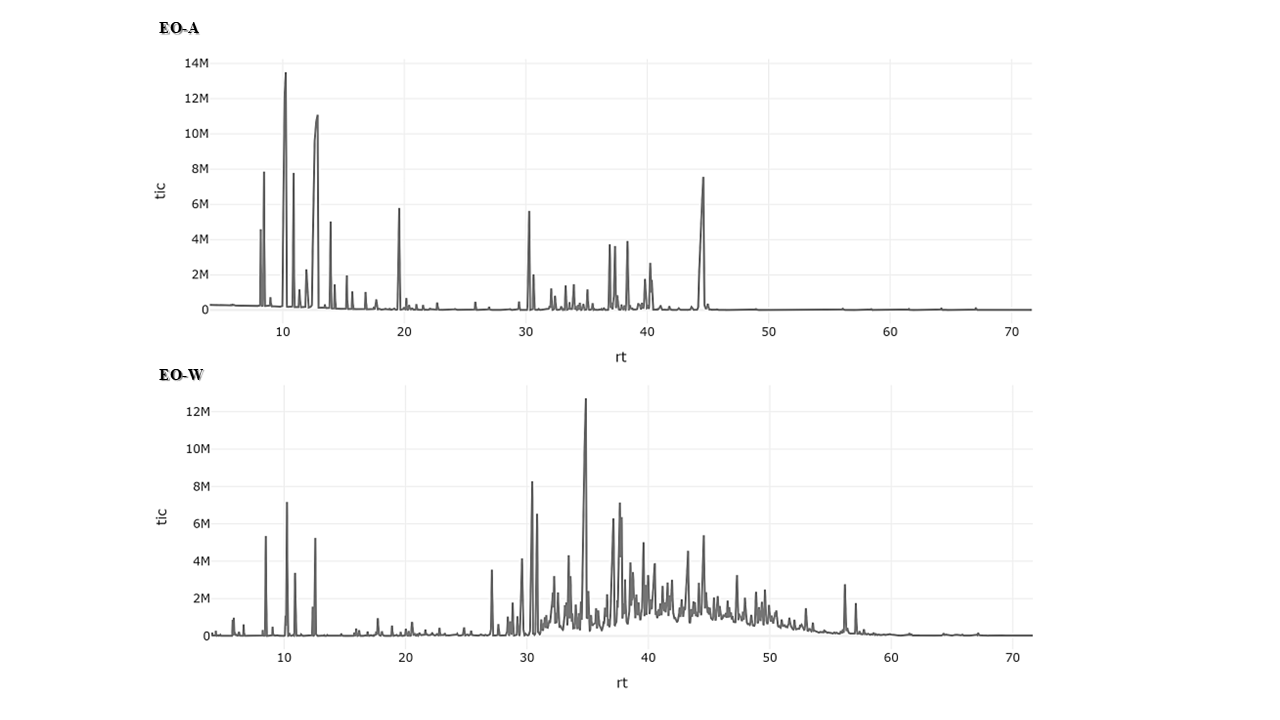
***Figure S1.*** *GC-MS total ion chromatograms of essential oils from D. lanceolata leaves. (EO-A and EO-W)*


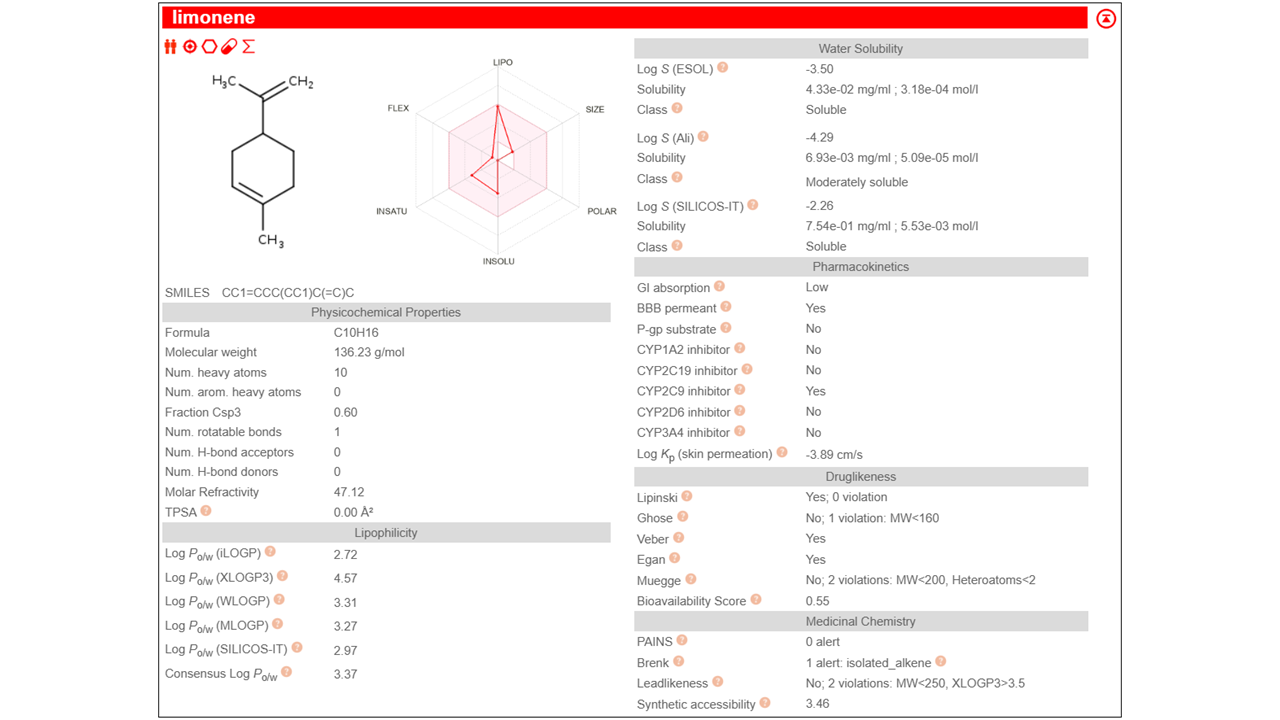
***Figure S2.*** *In silico ADME/T profile of limonene predicted by SwissADME, including physicochemical properties, drug-likeness filters, lipophilicity, solubility, and cytochrome P450 inhibition probabilities.*


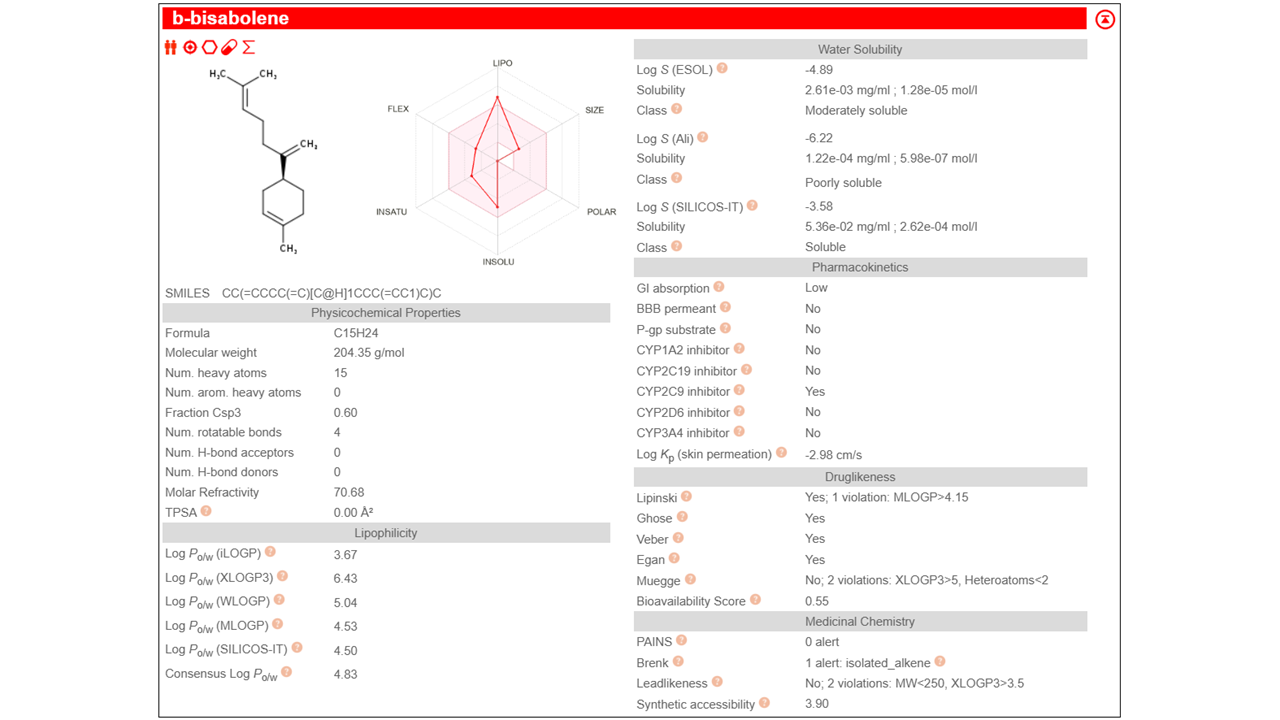
***Figure S3.*** *In silico ADME/T profile of β-bisabolene predicted by SwissADME, including physicochemical properties, drug-likeness filters, lipophilicity, solubility, and cytochrome P450 interaction probabilities.*


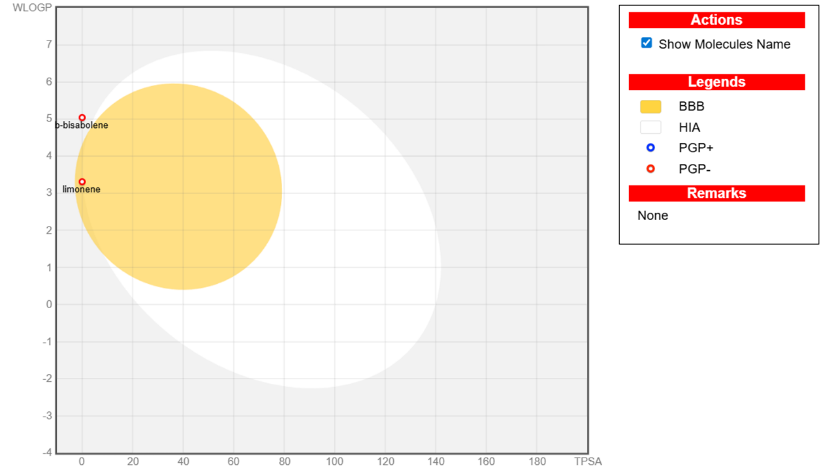
 ***Figure S4.*** *“Boiled-Egg” model illustrating the predicted gastrointestinal absorption and blood–brain barrier (BBB) permeation of limonene and β-bisabolene. The yolk (yellow) represents the region of high BBB permeation probability, while the white region corresponds to high intestinal absorption. The positions of the two terpenes highlight distinct distribution profiles, with limonene located within the BBB-permeant region and β-bisabolene positioned outside this zone, indicating restricted central nervous system access.*

*
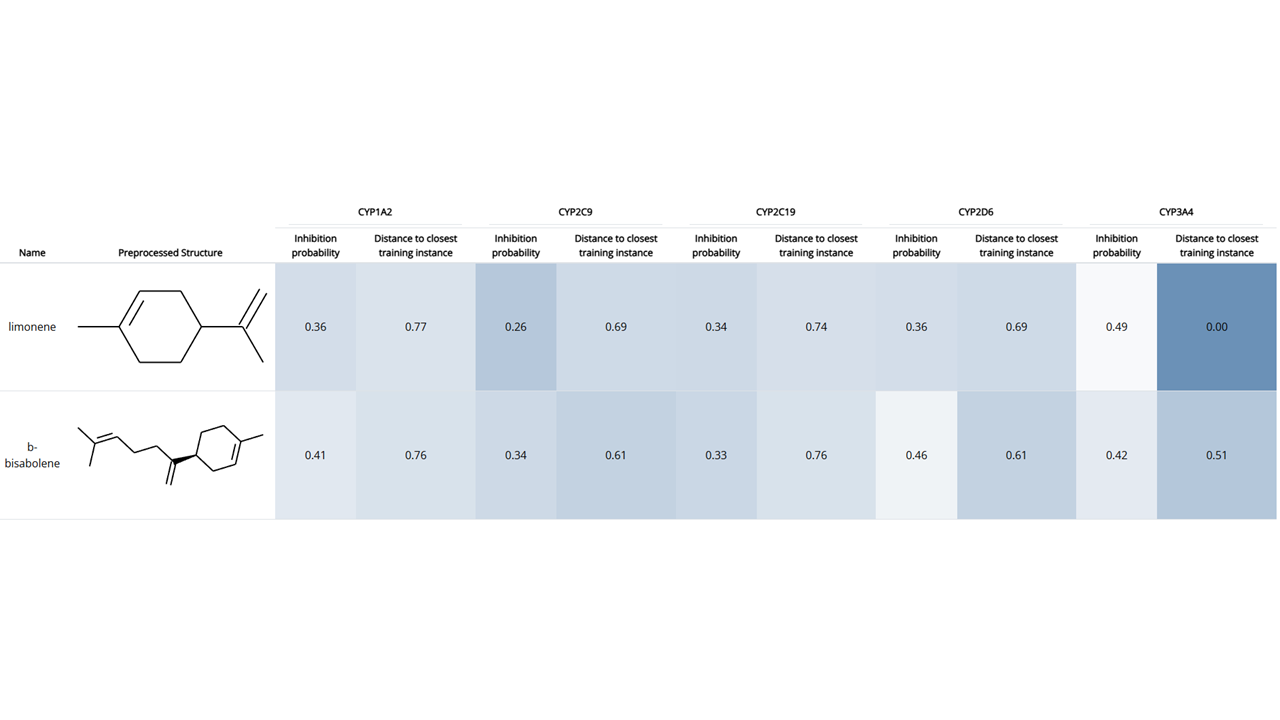
****Figure S5.*** *CYPlebrity radar plot showing the predicted interaction of limonene and β-bisabolene with major human cytochrome P450 isoforms, indicating a generally low to moderate inhibition potential and a favorable metabolic safety profile.*

***Table S1.*** *Chemical composition of the essential oil of D. lanceolata (EO-A and EO-W) - List of annotated compounds by GNPS.*

| **S** | **P** | **RT** | **Compound** | **RI** | **MW** | **Cos** | **LC** | **Conc.%** | |
| --- | --- | --- | --- | --- | --- | --- | --- | --- | --- |
|  |  |  |  |  |  |  |  | **EO-A** | **EO-W** |
| 9 | 1 | 8.19 | α-terpinene | 1017 | 136 | 0.86 | Gold | 1.41 | 0.09 |
| 10 | 2 | 8.44 | γ-terpinene | 1060 | 136 | 0.85 | Gold | 2.83 | 2.04 |
| 11 | 3 | 8.99 | β-(Z)-ocimene | 1037 | 136 | 0.70 | Gold | 0.17 | 0.00 |
| 14 | 4 | 10.1 | sabinene | 975 | 136 | 0.88 | Gold | 0.00 | 0.19 |
| 15 | 5 | 10.22 | α-pinene | 939 | 136 | 0.82 | Gold | 15.06 | 2.78 |
| 18 | 6 | 10.88 | β-pinene | 979 | 136 | 0.90 | Gold | 2.82 | 1.33 |
| 20 | 7 | 11.94 | terpinolene | 1088 | 136 | 0.70 | - | 1.35 | 0.00 |
| 22 | 8 | 12.56 | dihydrocarveol | 1193 | 154 | 0.89 | Gold | 0.00 | 3.26 |
| 23 | 9 | 12.85 | limonene | 1029 | 166 | 0.70 | Gold | 28.53 | 0.00 |
| 25 | 10 | 13.93 | iso-3-thujanol | 1146 | 154 | 0.91 | Gold | 1.79 | 0.00 |
| 26 | 11 | 14.28 | β-(E)terpineol | 1165 | 154 | 0.78 | Gold | 0.45 | 0.00 |
| 28 | 12 | 15.27 | α-ionone | 1426 | 192 | 0.86 | Gold | 0.61 | 0.00 |
| 29 | 13 | 15.72 | terpinen-4-ol | 1177 | 154 | 0.70 | Gold | 0.34 | 0.00 |
| 31 | 14 | 15.93 | linalool | 1097 | 154 | 0.94 | Gold | 0.00 | 0.12 |
| 32 | 15 | 16.82 | (E)-pulegol | 1186 | 154 | 0.70 | - | 0.38 | 0.09 |
| 36 | 16 | 17.71 | (Z)-pulegol | 1192 | 154 | 0.70 | - | 0.23 | 0.37 |
| 39 | 17 | 19.57 | 4-carvomenthenol | 1177 | 154 | 0.94 | Gold | 3.09 | 0.06 |
| 42 | 18 | 20.18 | α-terpineol | 1189 | 154 | 0.96 | Gold | 0.25 | 0.00 |
| 45 | 19 | 20.52 | 2-carene | 1002 | 136 | 0.87 | Gold | 0.00 | 0.43 |
| 54 | 20 | 27.1 | γ-elemene | 1334 | 204 | 0.79 | Gold | 0.01 | 1.55 |
| 55 | 21 | 27.64 | α-muurolene | 1499 | 204 | 0.79 | Gold | 0.01 | 0.21 |
| 57 | 22 | 28.62 | eremorphilene | 1480 | 204 | 0.84 | Gold | 0.02 | 0.31 |
| 58 | 23 | 28.82 | α-cubebene | 1351 | 204 | 0.92 | Gold | 0.00 | 0.8 |
| 59 | 24 | 29.45 | β-(Z)-farnesene | 1443 | 204 | 0.70 | - | 0.23 | 0.09 |
| 62 | 25 | 29.59 | isocaryophyllene | 1408 | 204 | 0.73 | Gold | 0.00 | 1.8 |
| 63 | 26 | 30.27 | 6,9-guaiadiene | 1440 | 204 | 0.79 | Gold | 3.15 | 0.00 |
| 64 | 27 | 30.43 | β-gurjunene | 1432 | 204 | 0.77 | Gold | 0.01 | 6.39 |
| 65 | 28 | 30.64 | isocaryophyllene | 1408 | 204 | 0.86 | Gold | 0.90 | 0.00 |
| 66 | 29 | 30.82 | β-caryophyllene | 1419 | 220 | 0.70 | Gold | 0.00 | 4.72 |
| 67 | 30 | 31.18 | γ-muurolene | 1477 | 204 | 0.92 | Gold | 0.00 | 0.23 |
| 68 | 31 | 31.47 | α-cedrene | 1412 | 204 | 0.93 | Gold | 0.01 | 0.16 |
| 69 | 32 | 31.59 | aromadendrene | 1441 | 204 | 0.79 | Gold | 0.01 | 0.26 |
| 72 | 33 | 31.98 | (E)-cycloisolongifol-5-ol | 1555 | 220 | 0.70 | - | 0.15 | 0.00 |
| 74 | 34 | 32.1 | β-vetivenene | 1515 | 202 | 0.70 | Gold | 0.54 | 0.34 |
| 75 | 35 | 32.1 | unknown | - | 204 | 0.70 | - | 0.01 | 0.01 |
| 76 | 36 | 32.24 | α-humulene | 1454 | 204 | 0.86 | Gold | 0.01 | 0.84 |
| 77 | 37 | 32.41 | (Z)-nerolidol | 1533 | 222 | 0.77 | Gold | 0.01 | 0.01 |
| 78 | 38 | 32.41 | β-(E)-farnesene | 1457 | 204 | 0.90 | Gold | 0.39 | 0.20 |
| 79 | 39 | 32.55 | α-cadinene | 1538 | 204 | 0.70 | - | 0.00 | 1.08 |
| 80 | 40 | 32.83 | α-(E,E)-farnesene | 1506 | 204 | 0.70 | Gold | 0.13 | 0.03 |
| 84 | 41 | 33.12 | β-copaen-4-α-ol | 1590 | 220 | 0.70 | - | 0.04 | 0.30 |
| 85 | 42 | 33.28 | γ-cadinene | 1513 | 204 | 0.77 | Gold | 0.57 | 0.43 |
| 87 | 43 | 33.42 | δ-cadinene | 1523 | 204 | 0.70 | - | 0.00 | 1.89 |
| 88 | 44 | 33.58 | β-sesquiphellandrene | 1522 | 204 | 0.82 | Gold | 0.21 | 1.15 |
| 89 | 45 | 33.73 | α-cadinene | 1538 | 204 | 0.74 | Gold | 0.05 | 0.22 |
| 92 | 46 | 33.96 | β-germacrene | 1485 | 204 | 0.88 | Gold | 0.78 | 0.0 |
| 94 | 48 | 34.02 | (Z)-muurol-5-en-4-α-ol | 1572 | 222 | 0.82 | Gold | 0.00 | 0.56 |
| 97 | 49 | 34.25 | carotol | 1594 | 222 | 0.75 | Gold | 0.13 | 0.50 |
| 98 | 50 | 34.45 | bisabol-11-ol | 1590 | 222 | 0.70 | Gold | 0.17 | 0.60 |
| 100 | 51 | 34.62 | allo-aromadendrene epoxide | 1658 | 220 | 0.71 | Gold | 0.23 | 0.00 |
| 102 | 52 | 34.83 | β-bisabolene | 1509 | 204 | 0.90 | Gold | 0.00 | 16.45 |
| 103 | 53 | 34.93 | guaiol | 1600 | 136 | 0.86 | Gold | 0.01 | 0.00 |
| 106 | 55 | 35.08 | Styrene <2,4,6-trimethoxy-> | 1563 | 204 | 0.85 | Gold | 0.53 | 0.81 |
| 108 | 56 | 35.27 | unknown | - | 222 | 0.70 | - | 0.01 | 0.20 |
| 110 | 57 | 35.69 | occidentalol acetate | 1650 | 262 | 0.70 | Gold | 0.00 | 0.43 |
| 111 | 58 | 35.87 | germacrone | 1693 | 218 | 0.74 | Gold | 0.02 | 0.42 |
| 113 | 59 | 36.31 | elemol | 1550 | 222 | 0.70 | Gold | 0.02 | 0.07 |
| 114 | 60 | 36.42 | (Z,E)-farnesol | 1722 | 222 | 0.70 | Gold | 0.07 | 0.26 |
| 117 | 61 | 36.6 | γ-elemene | 1334 | 204 | 0.82 | Gold | 0.00 | 0.59 |
| 118 | 62 | 36.73 | unknown | - | 220 | 0.70 | - | 0.01 | 0.13 |
| 121 | 63 | 36.84 | α-corocalene | 1630 | 200 | 0.70 | Gold | 0.00 | 0.55 |
| 122 | 64 | 36.92 | cuprenen-1-ol <4-> | 1640 | 222 | 0.70 | Gold | 2.06 | 0.01 |
| 130 | 66 | 37.34 | khusinol | 1650 | 220 | 0.81 | Gold | 2.23 | 0.00 |
| 131 | 67 | 37.42 | (Z)-asarone | 1621 | 208 | 0.70 | - | 0.00 | 0.22 |
| 132 | 68 | 37.52 | caryophyllene oxide | 1583 | 220 | 0.87 | Gold | 0.61 | 0.00 |
| 134 | 70 | 37.64 | spathulenol | 1578 | 220 | 0.70 | - | 0.00 | 2.43 |
| 135 | 71 | 37.71 | 14-hydroxy-(Z)-caryophyllene | 1660 | 220 | 0.87 | Gold | 0.10 | 0.00 |
| 136 | 72 | 37.71 | 14-hydroxy-9-epi-(E)-caryophyllene | 1668 | 220 | 0.70 | Gold | 0.10 | 0.00 |
| 138 | 73 | 37.78 | gymnomitrol | 1600 | 220 | 0.70 | Gold | 0.21 | 2.05 |
| 140 | 74 | 38.07 | α-bisabolol acetate | 1720 | 264 | 0.73 | Gold | 0.15 | 0.74 |
| 143 | 75 | 38.38 | α-selinene | 1498 | 204 | 0.78 | Gold | 2.44 | 0.00 |
| 145 | 76 | 38.52 | longifolol | 1590 | 222 | 0.76 | Gold | 0.00 | 1.65 |
| 147 | 77 | 38.72 | bisabolone oxide | 1597 | 236 | 0.73 | Gold | 0.00 | 0.73 |
| 148 | 78 | 38.83 | unknown | - | 290 | 0.70 | - | 0.02 | 0.12 |
| 149 | 79 | 39.01 | unknown | - | 162 | 0.70 | - | 0.02 | 0.36 |
| 151 | 80 | 39.08 | unknown | - | 220 | 0.70 | - | 0.02 | 0.00 |
| 152 | 81 | 39.19 | α-cyperone | 1750 | 218 | 0.75 | Gold | 0.29 | 0.26 |
| 156 | 82 | 39.59 | (Z)-bergamotol | 1680 | 220 | 0.70 | Gold | 0.24 | 2.12 |
| 157 | 83 | 39.77 | cedrane | 1390 | 206 | 0.70 | Gold | 1.30 | 0.99 |
| 159 | 84 | 39.92 | α-bisabolene | 1504 | 204 | 0.70 | Gold | 0.00 | 1.51 |
| 160 | 85 | 40.02 | curcumenol | 1650 | 234 | 0.70 | Gold | 0.19 | 0.00 |
| 161 | 86 | 40.02 | guaiol acetate | 1715 | 264 | 0.70 | - | 0.01 | 0.00 |
| 164 | 87 | 40.13 | β-chenopodiol | 1550 | 238 | 0.74 | Gold | 1.58 | 0.15 |
| 167 | 88 | 40.38 | β-eudesmol | 1651 | 222 | 0.78 | Gold | 0.89 | 0.25 |
| 169 | 89 | 40.44 | α-muurolol | 1646 | 222 | 0.70 | - | 0.00 | 0.10 |
| 170 | 90 | 40.52 | cedranyl acetate | 1675 | 264 | 0.70 | Gold | 0.00 | 0.27 |
| 174 | 91 | 40.7 | α-costol | 1762 | 220 | 0.70 | - | 0.02 | 0.07 |
| 177 | 92 | 40.83 | unknown | - | 220 | 0.70 | - | 0.03 | 0.05 |
| 178 | 93 | 40.97 | unknown | - | 218 | 0.70 | - | 0.00 | 0.23 |
| 180 | 94 | 41.17 | α-sinensal | 1758 | 218 | 0.83 | Gold | 0.25 | 0.68 |
| 181 | 95 | 41.33 | carissone | 1815 | 236 | 0.70 | - | 0.02 | 0.17 |
| 184 | 97 | 41.58 | (E)-asarone | 1676 | 208 | 0.70 | Gold | 0.00 | 0.67 |
| 185 | 98 | 41.73 | β-atlantol | 1720 | 220 | 0.70 | Gold | 0.03 | 0.42 |
| 187 | 99 | 41.94 | β-bergamotene | 1436 | 204 | 0.70 | Gold | 0.02 | 0.45 |
| 189 | 100 | 42.57 | laurenene | 1370 | 272 | 0.70 | Gold | 0.09 | 0.18 |
| 190 | 101 | 42.74 | unknown | - | 220 | 0.72 | - | 0.01 | 0.43 |
| 191 | 102 | 42.93 | unknown | - | 220 | 0.70 | - | 0.02 | 0.14 |
| 194 | 103 | 43.71 | β-ionone | 1488 | 192 | 0.74 | Gold | 0.17 | 0.36 |
| 197 | 104 | 44.38 | khusimol | 1740 | 204 | 0.70 | Gold | 0.00 | 0.09 |
| 199 | 105 | 44.55 | cyclocolorenone | 1744 | 218 | 0.72 | Gold | 12.78 | 3.92 |
| 200 | 106 | 44.6 | cyclocolorenone <epi-> | 1750 | 218 | 0.73 | Gold | 0.02 | 0.34 |
| 204 | 107 | 45.05 | boronal | 1600 | 206 | 0.70 | Gold | 0.00 | 0.24 |
| 205 | 108 | 45.25 | unknown | - | 152 | 0.70 | - | 0.00 | 0.43 |
| 210 | 109 | 45.7 | n-hexadecanol | 1878 | 242 | 0.74 | Gold | 0.00 | 0.26 |
| 215 | 110 | 46.38 | hexadecyl acetate | 1985 | 284 | 0.70 | - | 0.00 | 0.05 |
| 219 | 111 | 47.29 | drimenin | 1745 | 234 | 0.70 | Gold | 0.00 | 1.51 |
| 223 | 112 | 47.95 | columellarin | 1700 | 232 | 0.70 | Gold | 0.00 | 0.01 |
| 224 | 113 | 47.95 | unknown | - | 164 | 0.74 | - | 0.00 | 0.59 |
| 226 | 114 | 48.53 | sinulariolide | 2150 | 334 | 0.70 | - | 0.00 | 0.14 |
| 227 | 115 | 48.87 | phyllocladene | 1968 | 272 | 0.70 | Gold | 0.03 | 1.00 |
| 228 | 116 | 49.11 | ugandensidial | 1700 | 308 | 0.70 | Gold | 0.00 | 0.32 |
| 229 | 117 | 49.34 | calamenene | 1515 | 202 | 0.70 | Gold | 0.00 | 0.67 |
| 230 | 118 | 49.6 | allethrin I | 2100 | 302 | 0.73 | Gold | 0.00 | 0.97 |
| 231 | 119 | 49.92 | allethrin II | 2250 | 346 | 0.80 | Gold | 0.00 | 0.30 |
| 234 | 120 | 50.45 | unknown | - | 228 | 0.70 | - | 0.00 | 0.03 |
| 236 | 121 | 51.61 | unknown | - | 256 | 0.70 | - | 0.00 | 0.14 |
| 237 | 122 | 52.02 | unknown | - | 225 | 0.70 | - | 0.00 | 0.19 |
| 239 | 123 | 52.97 | flexibilide | 2100 | 334 | 0.70 | Gold | 0.00 | 0.61 |
| 240 | 124 | 53.54 | n-octadecanol | 2077 | 270 | 0.70 | - | 0.00 | 0.16 |
| 242 | 125 | 56.18 | isomenthol | 1183 | 156 | 0.86 | Gold | 0.06 | 1.67 |
| 245 | 126 | 57.08 | (Z)-9-heptacosene | 2670 | 378 | 0.63 | Gold | 0.00 | 0.74 |
|  |  |  | **Natural Products Classes** |  |  |  |  | **Conc.%** | |
|  |  |  | *Monoterpenes* |  |  |  |  | 58.80 | 12.86 |
|  |  |  | *Sesquiterpenes* |  |  |  |  | 34.20 | 66.80 |
|  |  |  | Others |  |  |  |  | 0.76 | 7.84 |
|  |  |  | **Total composition** |  |  |  |  | **93.76** | **87.54** |

***S:*** *Scan;* ***P:*** *Peak;* ***RT:*** *Retention Time;* ***RI:*** *Retention Index;* ***MW:*** *Molecular Weight;* ***LC:*** *Library Class*

**Table S2.** Statistical analysis – ANOVA: Disk Diffusion Method - Zone of inibition (cm)

| **Disk Diffusion Method - Zone of inibition (cm)** | | | | | | | | | | | | | | | |  |
| --- | --- | --- | --- | --- | --- | --- | --- | --- | --- | --- | --- | --- | --- | --- | --- | --- |
|  |  |  | |  |  | |  | |  | | |  | | Shapiro-Wilk | |  |
| **Species (Lineage)** | **Code** | **N** | **Mean** | | | **SE** | | **SD** | | **Minimum** | **Maximum** | | **W** | | **p** | |
| *Staphylococcus aureus* (ATCC 25923) | **EOA** | 3 | 0.700 | | | 0.0000 | | 0.0000 | | 0.700 | 0.700 | | NaN | | NaN | |
|  | **EOW** | 3 | 0.700 | | | 0.0000 | | 0.0000 | | 0.700 | 0.700 | | NaN | | NaN | |
|  | **(+)** ^a^ | 3 | 2.500 | | | 0.0577 | | 0.1000 | | 2.400 | 2.600 | | 1.000 | | 1.000 | |
| *Acinetobacter baumannii* (ATCC 19606) | **EOA** | 3 | 1.230 | | | 0.1039 | | 0.1800 | | 1.050 | 1.410 | | 1.000 | | 1.000 | |
|  | **EOW** | 3 | 0.000 | | | 0.0000 | | 0.0000 | | 0.000 | 0.000 | | NaN | | NaN | |
|  | **(+)** ^a^ | 3 | 2.170 | | | 0.0693 | | 0.1200 | | 2.050 | 2.290 | | 1.000 | | 1.000 | |
| *Saccharomyces cerevisiae* (BY4647) | **EOA** | 3 | 1.200 | | | 0.0000 | | 0.0000 | | 1.200 | 1.200 | | NaN | | NaN | |
|  | **EOW** | 3 | 0.930 | | | 0.0751 | | 0.1300 | | 0.800 | 1.060 | | 1.000 | | 1.000 | |
|  | **(+)** ^b^ | 3 | 2.070 | | | 0.0693 | | 0.1200 | | 1.950 | 2.190 | | 1.000 | | 1.000 | |
| *Cryptococcus neoformans* (KN99α serotype A) | **EOA** | 3 | 0.870 | | | 0.0404 | | 0.0700 | | 0.800 | 0.940 | | 1.000 | | 1.000 | |
|  | **EOW** | 3 | 0.830 | | | 0.0289 | | 0.0500 | | 0.780 | 0.880 | | 1.000 | | 1.000 | |
|  | **(+)** ^b^ | 3 | 1.700 | | | 0.0577 | | 0.1000 | | 1.600 | 1.800 | | 1.000 | | 1.000 | |
| *Cryptococcus neoformans* (JEC21 serotype D) | **EOA** | 3 | 0.830 | | | 0.0462 | | 0.0800 | | 0.750 | 0.910 | | 1.000 | | 1.000 | |
|  | **EOW** | 3 | 0.800 | | | 0.0577 | | 0.1000 | | 0.700 | 0.900 | | 1.000 | | 1.000 | |
|  | **(+)** ^b^ | 3 | 1.970 | | | 0.0693 | | 0.1200 | | 1.850 | 2.090 | | 1.000 | | 1.000 | |
| *Cryptococcus gattii* (R265 serotype B) | **EOA** | 3 | 0.800 | | | 0.0000 | | 0.0000 | | 0.800 | 0.800 | | NaN | | NaN | |
|  | **EOW** | 3 | 1.000 | | | 0.0000 | | 0.0000 | | 1.000 | 1.000 | | NaN | | NaN | |
|  | **(+)** ^b^ | 3 | 1.900 | | | 0.0000 | | 0.0000 | | 1.900 | 1.900 | | NaN | | NaN | |
| *Cryptococcus gattii* (NIH312 serotype C) | **EOA** | 3 | 0.900 | | | 0.0577 | | 0.1000 | | 0.800 | 1.000 | | 1.000 | | 1.000 | |
|  | **EOW** | 3 | 1.030 | | | 0.0346 | | 0.0600 | | 0.970 | 1.090 | | 1.000 | | 1.000 | |
|  | **(+)** ^b^ | 3 | 2.600 | | | 0.1443 | | 0.2500 | | 2.350 | 2.850 | | 1.000 | | 1.000 | |
| *Candida krusei* (Clinical isolate 9602) | **EOA** | 3 | 1.100 | | | 0.0577 | | 0.1000 | | 1.000 | 1.200 | | 1.000 | | 1.000 | |
|  | **EOW** | 3 | 0.000 | | | 0.0000 | | 0.0000 | | 0.000 | 0.000 | | NaN | | NaN | |
|  | **(+)** ^b^ | 3 | 2.090 | | | 0.1732 | | 0.3000 | | 1.790 | 2.390 | | 1.000 | | 1.000 | |
| *Candida parapsilosis* (Clinical isolate 68) | **EOA** | 3 | 0.770 | | | 0.0693 | | 0.1200 | | 0.650 | 0.890 | | 1.000 | | 1.000 | |
|  | **EOW** | 3 | 0.000 | | | 0.0000 | | 0.0000 | | 0.000 | 0.000 | | NaN | | NaN | |
|  | **(+)** ^c^ | 3 | 1.500 | | | 0.0000 | | 0.0000 | | 1.500 | 1.500 | | NaN | | NaN | |

***Positive control (+): ^a^****kanamicin;* ***^b^****eugenol;* ***^c^****amphotericin-b;* ***NaN:*** *Not a Number*

**Table S3.** One-Way ANOVA (Welch's)

| One-Way ANOVA (Welch's) | | | | |
| --- | --- | --- | --- | --- |
| Species (Lineage) | **Zone of inibition (cm)** | | | |
|  | **F** | **df1** | **df2** | **p** |
| *Staphylococcus aureus* (ATCC 25923) | NaN | 2 | NaN | NaN |
| *Acinetobacter baumannii* (ATCC 19606) | NaN | 2 | NaN | NaN |
| *Saccharomyces cerevisiae* (BY4647) | NaN | 2 | NaN | NaN |
| *Cryptococcus neoformans* (KN99α serotype A) | 80.4 | 2 | 3.74 | 8.48e-4 |
| *Cryptococcus neoformans* (JEC21 serotype D) | NaN | 2 | NaN | NaN |
| *Cryptococcus gattii* (R265 serotype B) | NaN | 2 | NaN | NaN |
| *Cryptococcus gattii* (NIH312 serotype C) | 51.1 | 2 | 3.41 | 0.00286 |
| *Candida krusei* (Clinical isolate 9602) | NaN | 2 | NaN | NaN |
| *Candida parapsilosis* (Clinical isolate 68) | NaN | 2 | NaN | NaN |

**Table S4**. Homogeneity of Variances Test (Levene's)

| Homogeneity of Variances Test (Levene's) | | | | |
| --- | --- | --- | --- | --- |
| Species (Lineage) | **Zone of inibition (cm)** | | | |
|  | **F** | **df1** | **df2** | **p** |
| *Staphylococcus aureus* (ATCC 25923) | 4.00 | 2 | 6 | 0.0787 |
| *Acinetobacter baumannii* (ATCC 19606) | 2.15 | 2 | 6 | 0.197 |
| *Saccharomyces cerevisiae* (BY4647) | 2.01 | 2 | 6 | 0.215 |
| *Cryptococcus neoformans* (KN99α serotype A) | 0.437 | 2 | 6 | 0.665 |
| *Cryptococcus neoformans* (JEC21 serotype D) | 2.05 | 2 | 6 | 0.210 |
| *Cryptococcus gattii* (R265 serotype B) | NaN | 2 | 6 | NaN |
| *Cryptococcus gattii* (NIH312 serotype C) | 1.58 | 2 | 6 | 0.281 |
| *Candida krusei* (Clinical isolate 9602) | 2.80 | 2 | 6 | 0.138 |
| *Candida parapsilosis* (Clinical isolate 68) | 4.00 | 2 | 6 | 0.0787 |

**Table S5.** Post Hoc Tests: Tukey Post-Hoc Test

| Post Hoc Tests: Tukey Post-Hoc Test | | | | | |
| --- | --- | --- | --- | --- | --- |
| Species (Lineage) |  |  | **Zone of inibition (cm)** | | |
|  |  |  | **EOA** | **EOW** | **(+)** |
| *Staphylococcus aureus* (ATCC 25923) | **EOA** | **Mean difference** | — | 0.00 | -1.80*** |
|  |  | **p-value** | — | NaN | 8.12e-4 |
|  | **EOW** | **Mean difference** |  | — | -1.80*** |
|  |  | **p-value** |  | — | 8.12e-4 |
|  | **(+)** | **Mean difference** |  |  | — |
|  |  | **p-value** |  |  | — |
| *Acinetobacter baumannii* (ATCC 19606) | **EOA** | **Mean difference** | — | 1.23* | -0.940** |
|  |  | **p-value** | — | 0.0129 | 0.00605 |
|  | **EOW** | **Mean difference** |  | — | -2.170*** |
|  |  | **p-value** |  | — | 7.91e-4 |
|  | **(+)** | **Mean difference** |  |  | — |
|  |  | **p-value** |  |  | — |
| *Saccharomyces cerevisiae* (BY4647) | **EOA** | **Mean difference** | — | 0.270 | -0.870* |
|  |  | **p-value** | — | 0.124 | 0.0115 |
|  | **EOW** | **Mean difference** |  | — | -1.140*** |
|  |  | **p-value** |  | — | 8.44e-4 |
|  | **(+)** | **Mean difference** |  |  | — |
|  |  | **p-value** |  |  | — |
| *Cryptococcus neoformans* (KN99α serotype A) | **EOA** | **Mean difference** | — | 0.0400 | -0.830** |
|  |  | **p-value** | — | 0.722 | 0.00118 |
|  | **EOW** | **Mean difference** |  | — | -0.870** |
|  |  | **p-value** |  | — | 0.00202 |
|  | **(+)** | **Mean difference** |  |  | — |
|  |  | **p-value** |  |  | — |
| *Cryptococcus neoformans* (JEC21 serotype D) | **EOA** | **Mean difference** | — | 0.0300 | -1.07** |
|  |  | **p-value** | — | 0.915 | 0.00285 |
|  | **EOW** | **Mean difference** |  | — | -1.10** |
|  |  | **p-value** |  | — | 0.00483 |
|  | **(+)** | **Mean difference** |  |  | — |
|  |  | **p-value** |  |  | — |
| *Cryptococcus gattii* (R265 serotype B) | **EOA** | **Mean difference** | — | -0.200 | -1.100 |
|  |  | **p-value** | — | NaN | NaN |
|  | **EOW** | **Mean difference** |  | — | -0.900 |
|  |  | **p-value** |  | — | NaN |
|  | **(+)** | **Mean difference** |  |  | — |
|  |  | **p-value** |  |  | — |
| *Cryptococcus gattii* (NIH312 serotype C) | **EOA** | **Mean difference** | — | -0.130 | -1.70** |
|  |  | **p-value** | — | 0.265 | 0.00579 |
|  | **EOW** | **Mean difference** |  | — | -1.57* |
|  |  | **p-value** |  | — | 0.01129 |
|  | **(+)** | **Mean difference** |  |  | — |
|  |  | **p-value** |  |  | — |
| *Candida krusei* (Clinical isolate 9602) | **EOA** | **Mean difference** | — | 1.10** | -0.990* |
|  |  | **p-value** | — | 0.00483 | 0.0394 |
|  | **EOW** | **Mean difference** |  | — | -2.090* |
|  |  | **p-value** |  | — | 0.0124 |
|  | **(+)** | **Mean difference** |  |  | — |
|  |  | **p-value** |  |  | — |
| *Candida parapsilosis* (Clinical isolate 68) | **EOA** | **Mean difference** | — | 0.770* | -0.730* |
|  |  | **p-value** | — | 0.0146 | 0.0162 |
|  | **EOW** | **Mean difference** |  | — | -1.500 |
|  |  | **p-value** |  | — | NaN |
|  | **(+)** | **Mean difference** |  |  | — |
|  |  | **p-value** |  |  | — |
| *Note. * p < .05, ** p < .01, *** p < .001* |  |  |  |  |  |

**Table S6.** Statistical analysis – ANOVA: MIC_90_

| **Minimum inhibitory concentrations - (mg.mL^-1^)** | | | | |
| --- | --- | --- | --- | --- |
| **Species (Lineage)** | **Code** | **N** | **MIC_90_** | **SD** |
| *Staphylococcus aureus* (ATCC 25923) | **EOA** | 3 | 0.270 | 0.00 |
|  | **EOW** | 3 | 0.270 | 0.00 |
|  | **(+)** ^a^ | 3 | 0.008 | 0.00 |
| *Acinetobacter baumannii* (ATCC 19606) | **EOA** | 3 | 0.135 | 0.00 |
|  | **EOW** | 3 | ≥ 0.400 | 0.00 |
|  | **(+)** **^b^** | 3 | 0.008 | 0.00 |
| *Saccharomyces cerevisiae* (BY4647) | **EOA** | 3 | 0.135 | 0.00 |
|  | **EOW** | 3 | 0.033 | 0.00 |
|  | **(+)** **^c^** | 3 | 0.013 | 0.00 |
| *Cryptococcus neoformans* (KN99α serotype A) | **EOA** | 3 | 0.067 | 0.00 |
|  | **EOW** | 3 | 0.135 | 0.00 |
|  | **(+)** **^c^** | 3 | 0.013 | 0.00 |
| *Cryptococcus neoformans* (JEC21 serotype D) | **EOA** | 3 | 0.033 | 0.00 |
|  | **EOW** | 3 | 0.067 | 0.00 |
|  | **(+)** **^c^** | 3 | 0.006 | 0.00 |
| *Cryptococcus gattii* (R265 serotype B) | **EOA** | 3 | 0.016 | 0.00 |
|  | **EOW** | 3 | 0.067 | 0.00 |
|  | **(+)** **^c^** | 3 | 0.025 | 0.00 |
| *Cryptococcus gattii* (NIH312 serotype C) | **EOA** | 3 | 0.016 | 0.00 |
|  | **EOW** | 3 | 0.067 | 0.00 |
|  | **(+)** **^c^** | 3 | 0.006 | 0.00 |
| *Candida krusei* (Clinical isolate 9602) | **EOA** | 3 | 0.016 | 0.00 |
|  | **EOW** | 3 | ≥ 0.400 | 0.00 |
|  | **(+)** **^c^** | 3 | 0.050 | 0.00 |
| *Candida parapsilosis* (Clinical isolate 68) | **EOA** | 3 | 0.016 | 0.00 |
|  | **EOW** | 3 | ≥ 0.400 | 0.00 |
|  | **(+)** ^c^ | 3 | 0.006 | 0.00 |

***Positive control (+):*** *^a^vancomicin;* ***^b^****meropenem;* ***^c^****fluconazole.*

**Table S7.** One-Way ANOVA (Welch's)

| One-Way ANOVA (Welch's) | | | | |
| --- | --- | --- | --- | --- |
| Species (Lineage) | **MIC_90_** | | | |
|  | **F** | **df1** | **df2** | **p** |
| *Staphylococcus aureus* (ATCC 25923) | NaN | 2 | NaN | NaN |
| *Acinetobacter baumannii* (ATCC 19606) | NaN | 2 | NaN | NaN |
| *Saccharomyces cerevisiae* (BY4647) | NaN | 2 | NaN | NaN |
| *Cryptococcus neoformans* (KN99α serotype A) | NaN | 2 | NaN | NaN |
| *Cryptococcus neoformans* (JEC21 serotype D) | NaN | 2 | NaN | NaN |
| *Cryptococcus gattii* (R265 serotype B) | NaN | 2 | NaN | NaN |
| *Cryptococcus gattii* (NIH312 serotype C) | NaN | 2 | NaN | NaN |
| *Candida krusei* (Clinical isolate 9602) | NaN | 2 | NaN | NaN |
| *Candida parapsilosis* (Clinical isolate 68) | NaN | 2 | NaN | NaN |

**Table S8.** Post Hoc Tests: Tukey Post-Hoc Test

| Post Hoc Tests: Tukey Post-Hoc Test | | | | | |
| --- | --- | --- | --- | --- | --- |
| Species (Lineage) |  |  | **MIC_90_** | | |
|  |  |  | **EOA** | **EOW** | **(+)** |
| *Staphylococcus aureus* (ATCC 25923) | **EOA** | **Mean difference** | — | 0.00 | 0.262*** |
|  |  | **p-value** | — | NaN | 0.00 |
|  | **EOW** | **Mean difference** |  | — | 0.262*** |
|  |  | **p-value** |  | — | 0.00 |
|  | **(+)** | **Mean difference** |  |  | — |
|  |  | **p-value** |  |  | — |
| *Acinetobacter baumannii* (ATCC 19606) | **EOA** | **Mean difference** | — | -0.265*** | 0.127*** |
|  |  | **p-value** | — | 0.00 | 0.00 |
|  | **EOW** | **Mean difference** |  | — | 0.392*** |
|  |  | **p-value** |  | — | 0.00 |
|  | **(+)** | **Mean difference** |  |  | — |
|  |  | **p-value** |  |  | — |
| *Saccharomyces cerevisiae* (BY4647) | **EOA** | **Mean difference** | — | 0.102*** | 0.1220*** |
|  |  | **p-value** | — | 0.00 | 0.00 |
|  | **EOW** | **Mean difference** |  | — | 0.0200*** |
|  |  | **p-value** |  | — | 0.00 |
|  | **(+)** | **Mean difference** |  |  | — |
|  |  | **p-value** |  |  | — |
| *Cryptococcus neoformans* (KN99α serotype A) | **EOA** | **Mean difference** | — | -0.0680*** | 0.0540*** |
|  |  | **p-value** | — | 0.00 | 0.00 |
|  | **EOW** | **Mean difference** |  | — | 0.1220*** |
|  |  | **p-value** |  | — | 0.00 |
|  | **(+)** | **Mean difference** |  |  | — |
|  |  | **p-value** |  |  | — |
| *Cryptococcus neoformans* (JEC21 serotype D) | **EOA** | **Mean difference** | — | -0.0340*** | 0.0270*** |
|  |  | **p-value** | — | 0.00 | 0.00 |
|  | **EOW** | **Mean difference** |  | — | 0.0610*** |
|  |  | **p-value** |  | — | 0.00 |
|  | **(+)** | **Mean difference** |  |  | — |
|  |  | **p-value** |  |  | — |
| *Cryptococcus gattii* (R265 serotype B) | **EOA** | **Mean difference** | — | -0.0510*** | -0.00900*** |
|  |  | **p-value** | — | 0.00 | 0.00 |
|  | **EOW** | **Mean difference** |  | — | 0.04200*** |
|  |  | **p-value** |  | — | 0.00 |
|  | **(+)** | **Mean difference** |  |  | — |
|  |  | **p-value** |  |  | — |
| *Cryptococcus gattii* (NIH312 serotype C) | **EOA** | **Mean difference** | — | -0.0510*** | 0.0100*** |
|  |  | **p-value** | — | 0.00 | 0.00 |
|  | **EOW** | **Mean difference** |  | — | 0.0610*** |
|  |  | **p-value** |  | — | 0.00 |
|  | **(+)** | **Mean difference** |  |  | — |
|  |  | **p-value** |  |  | — |
| *Candida krusei* (Clinical isolate 9602) | **EOA** | **Mean difference** | — | -0.384*** | -0.0340*** |
|  |  | **p-value** | — | 0.00 | 0.00 |
|  | **EOW** | **Mean difference** |  | — | 0.3500*** |
|  |  | **p-value** |  | — | 0.00 |
|  | **(+)** | **Mean difference** |  |  | — |
|  |  | **p-value** |  |  | — |
| *Candida parapsilosis* (Clinical isolate 68) | **EOA** | **Mean difference** | — | -0.384*** | 0.0100*** |
|  |  | **p-value** | — | 0.00 | 0.00 |
|  | **EOW** | **Mean difference** |  | — | 0.3940*** |
|  |  | **p-value** |  | — | 0.00 |
|  | **(+)** | **Mean difference** |  |  | — |
|  |  | **p-value** |  |  | — |
| *Note. * p < .05, ** p < .01, *** p < .001* |  |  |  |  |  |
