# Supplementary figures and images for: GNPS Untargeted GC‐MS Metabolomic Analysis of Essential Oils From Duguetia lanceolata and Evaluation of Antimicrobial Activity
Source: Chem Biodivers. 2026 Feb 25;23(2):e03280. doi: 10.1002/cbdv.202503280 (PMC12935287; doi:10.1002/cbdv.202503280)

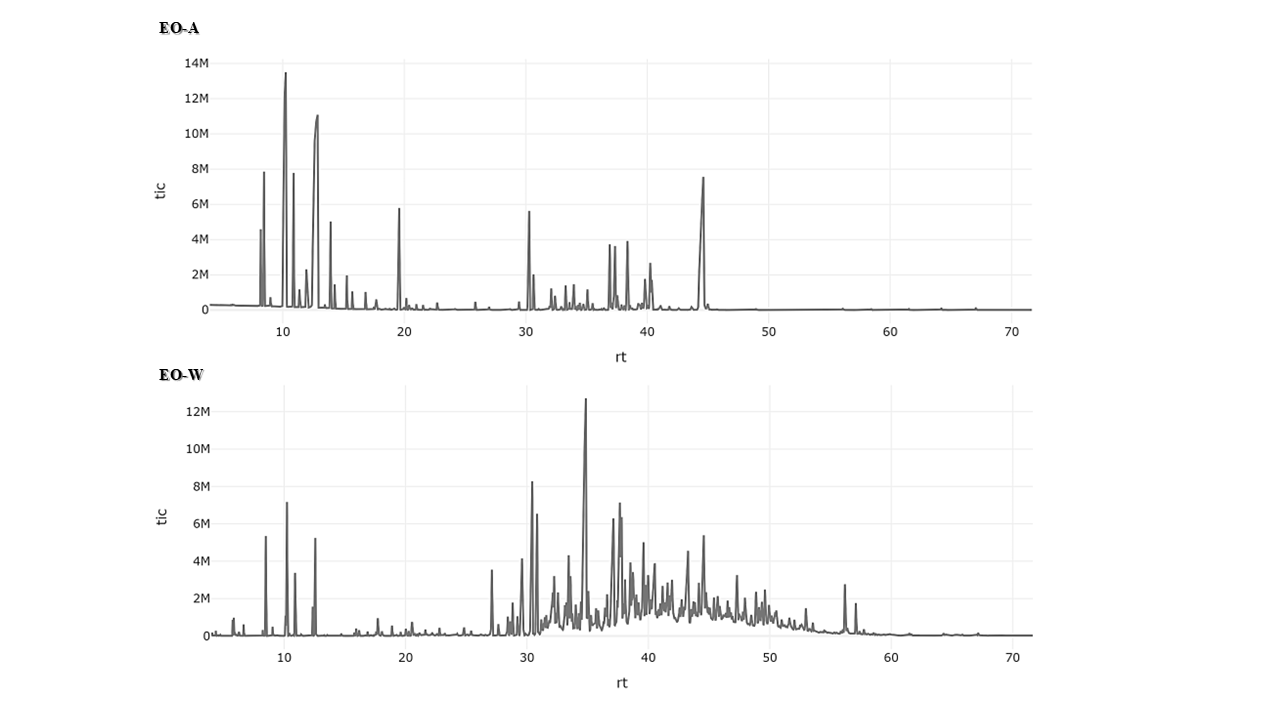

Supplement: Supplementary file 2 — Supporting File 2: cbdv70998‐sup‐0002‐FigureS1.TIF [file CBDV-23-e03280-s004.TIF]

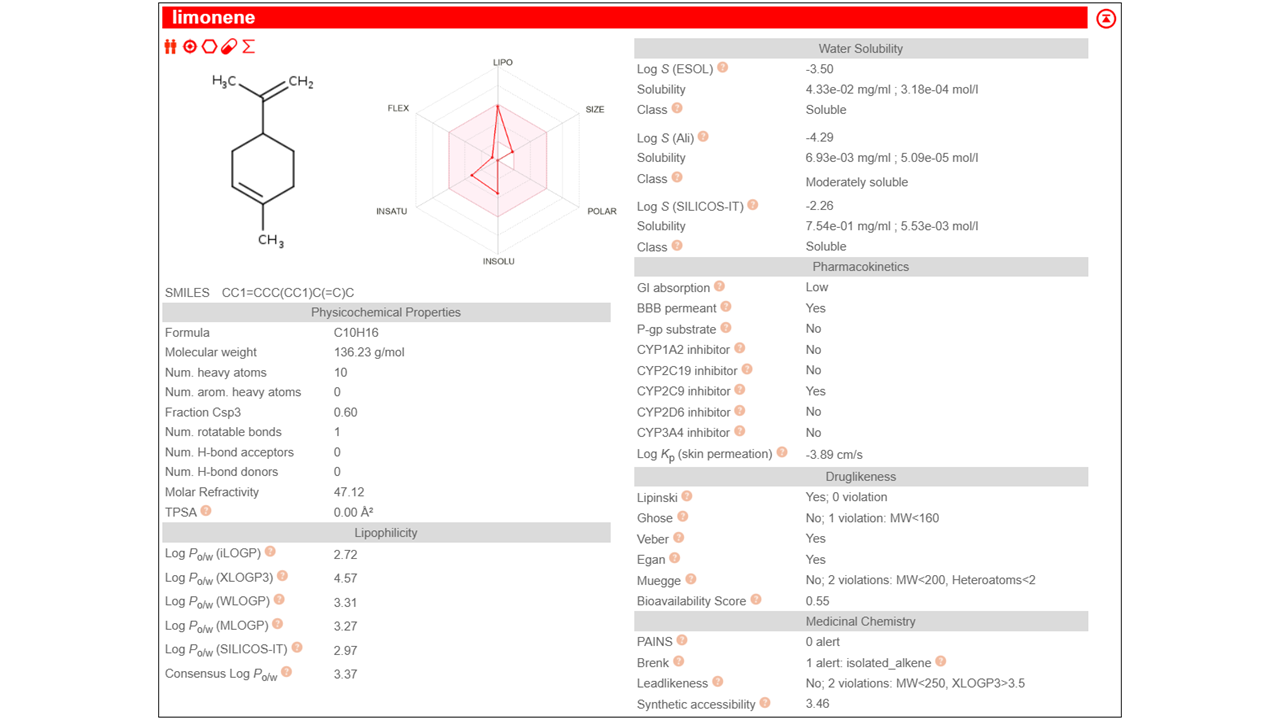

Supplement: Supplementary file 3 — Supporting File 3: cbdv70998‐sup‐0003‐FigureS2.TIF [file CBDV-23-e03280-s002.TIF]

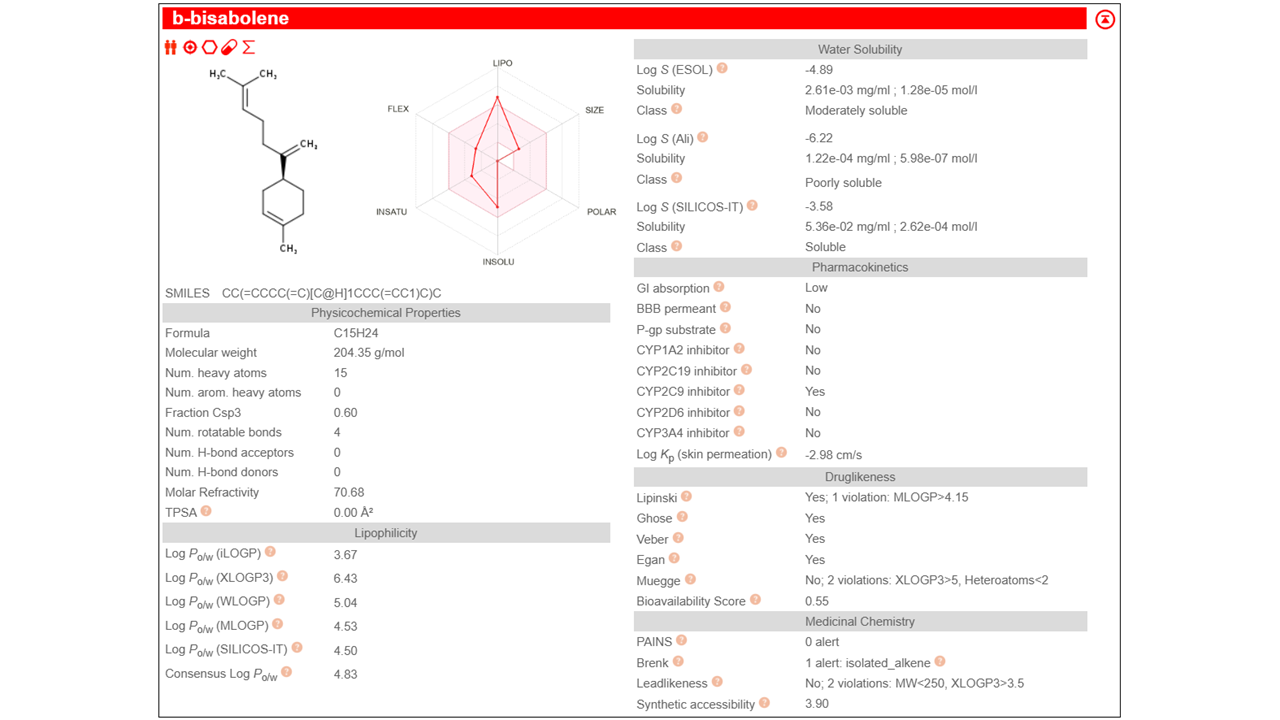

Supplement: Supplementary file 4 — Supporting File 4: cbdv70998‐sup‐0004‐FigureS3.TIF [file CBDV-23-e03280-s006.TIF]

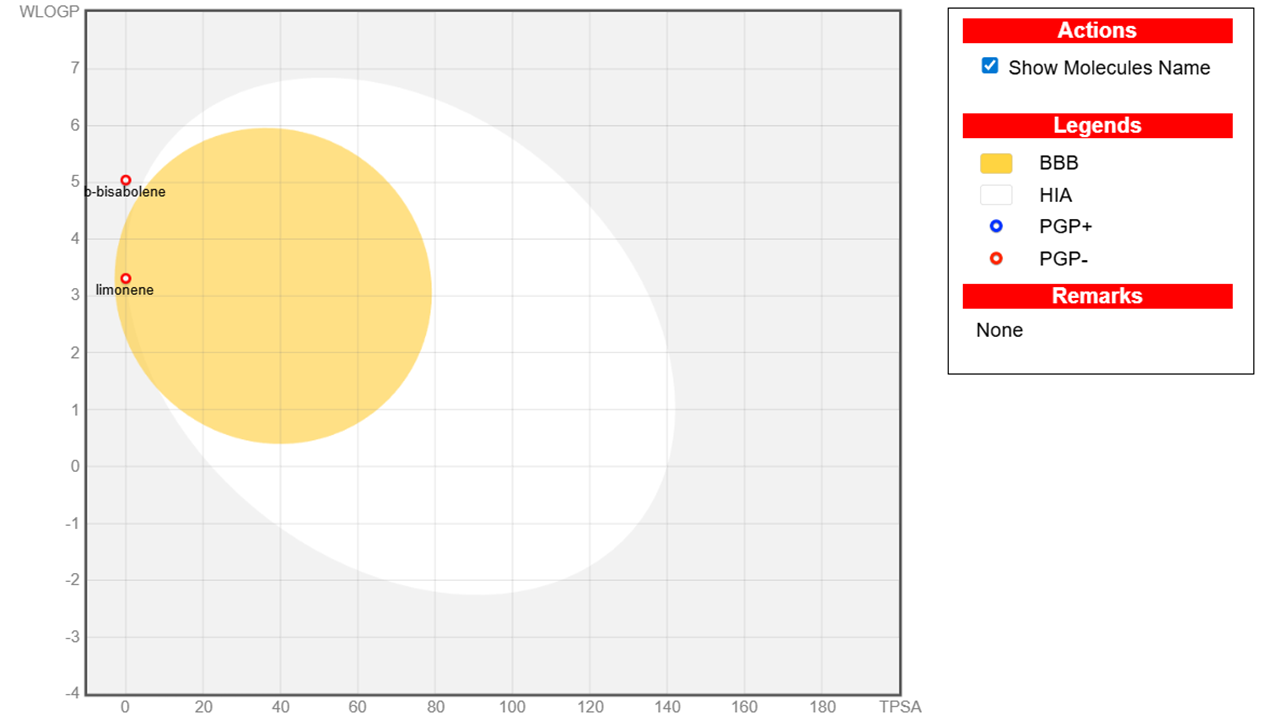

Supplement: Supplementary file 5 — Supporting File 5: cbdv70998‐sup‐0005‐FigureS4.TIF [file CBDV-23-e03280-s001.TIF]

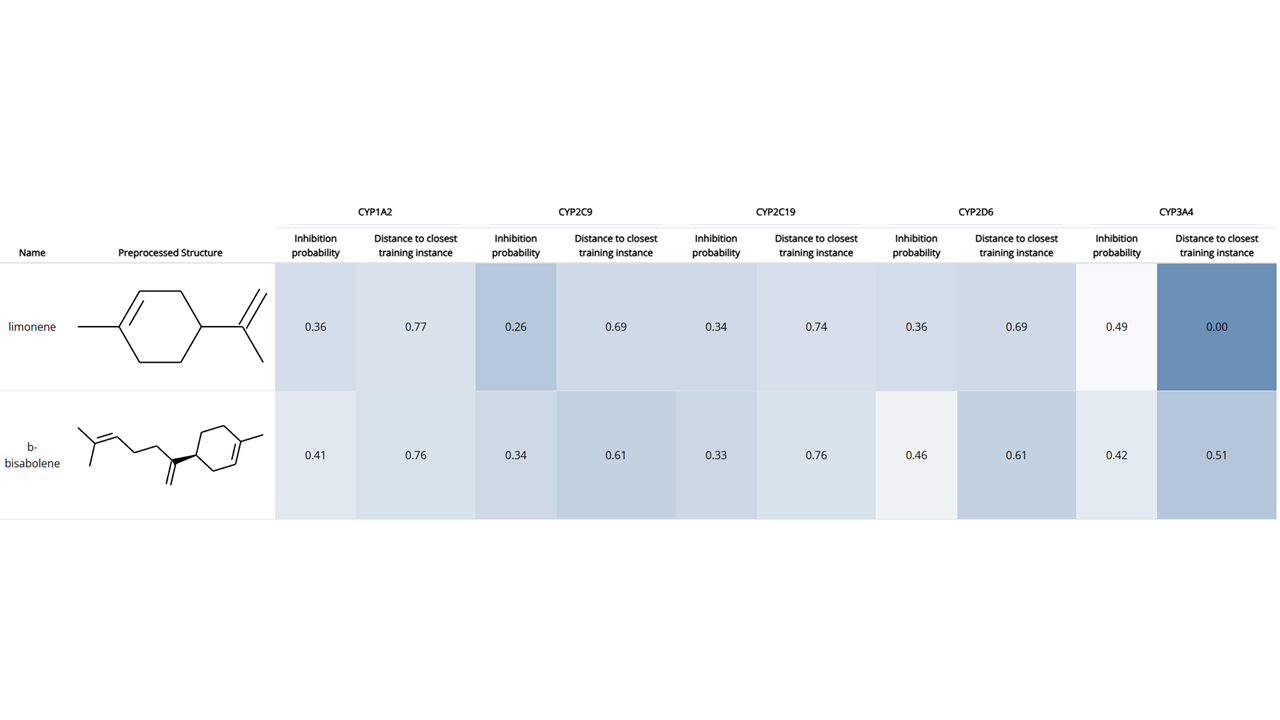

Supplement: Supplementary file 6 — Supporting File 6: cbdv70998‐sup‐0006‐FigureS5.tif [file CBDV-23-e03280-s005.tif]
